# EBAG9 controls CD8^+^ T cell memory formation responding to tumor challenge in mice

**DOI:** 10.1172/jci.insight.155534

**Published:** 2022-06-08

**Authors:** Armin Rehm, Anthea Wirges, Dana Hoser, Cornelius Fischer, Stefanie Herda, Kerstin Gerlach, Sascha Sauer, Gerald Willimsky, Uta E. Höpken

**Affiliations:** 1 Department of Translational Tumorimmunology, Max Delbrück Center for Molecular Medicine, Berlin, Germany.; 2 Institute of Immunology, Charité – Universitätsmedizin Berlin, corporate member of Freie Universität Berlin and Humboldt-Universität zu Berlin, Berlin Institute of Health, Berlin, Germany.; 3 German Cancer Research Center, Heidelberg, Germany.; 4 German Cancer Consortium, Berlin, Germany.; 5 Genomics Platform, Scientific Infrastructure Department, and; 6 Department of Microenvironmental Regulation in Autoimmunity and Cancer, Max Delbrück Center for Molecular Medicine, Berlin, Germany.

**Keywords:** Immunology, Oncology, Adaptive immunity, Leukemias, T cells

## Abstract

Insight into processes that determine CD8**^+^** T cell memory formation has been obtained from infection models. These models are biased toward an inflammatory milieu and often use high-avidity CD8**^+^** T cells in adoptive-transfer procedures. It is unclear whether these conditions mimic the differentiation processes of an endogenous repertoire that proceed upon noninflammatory conditions prevailing in premalignant tumor lesions. We examined the role of cytolytic capacity on CD8^+^ T cell fate decisions when primed by tumor cells or by minor histocompatibility antigen–mismatched leukocytes. CD8**^+^** memory commitment was analyzed in *Ebag9*-deficient mice that exhibited enhanced tumor cell lysis. This property endowed *Ebag9^–/–^* mice with extended control of Tcl-1 oncogene–induced chronic lymphocytic leukemia progression. In *Ebag9^–/–^* mice, an expanded memory population was obtained for anti-HY and anti–SV-40 T antigen–specific T cells, despite unchanged effector frequencies in the primary response. By comparing the single-cell transcriptomes of CD8^+^ T cells responding to tumor cell vaccination, we found differential distribution of subpopulations between *Ebag9^+/+^* and *Ebag9^–/–^* T cells. In *Ebag9^–/–^* cells, these larger clusters contained genes encoding transcription factors regulating memory cell differentiation and anti-apoptotic gene functions. Our findings link EBAG9-controlled cytolytic activity and the commitment to the CD8**^+^** memory lineage.

## Graphical abstract

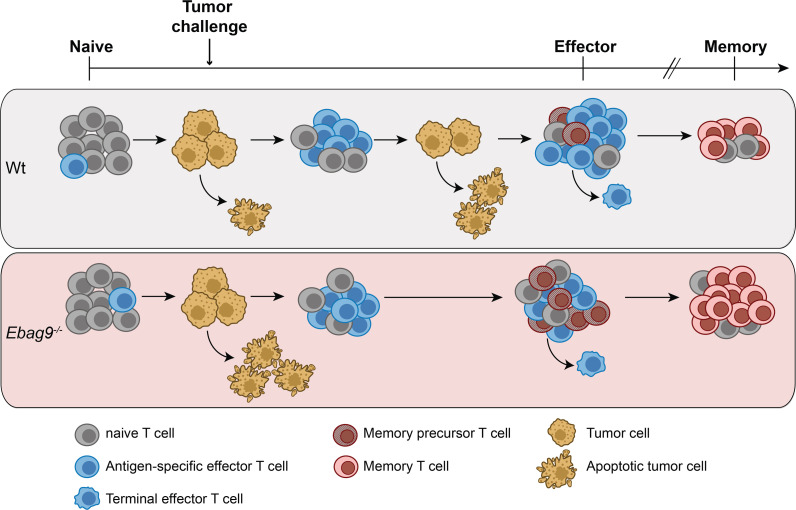

## Introduction

The cytolytic capacity of cytotoxic T lymphocytes (CTLs) is crucial for the ability to eliminate virus-infected and oncogene-transformed cells (1). Delivery of newly synthesized or recycled effector molecules like granzymes and perforin to the immunological synapse is a critical step in gaining CTL competence (2).

Upon acute infection or vaccination, naive T cells become activated and first differentiate into a pool of functional effector cells (KLRG1^hi^) and memory precursor cells (KLRG1^lo^CD127^hi^) (3). Although the KLRG1^hi^ effector cell population is terminal and largely disappears, the memory precursor subset is likely to mature into long-term memory (4). The pathways that drive the differential development of effector versus memory CTLs during antigen-specific clonal expansion under various conditions are not completely understood. Because development of vaccination strategies against infectious diseases is a leading motivation for studying mechanisms of memory differentiation, most models are biased toward a strong inflammatory milieu (5, 6). In principle, a short T cell receptor (TCR) stimulus in conjunction with a costimulatory signal induces a period of proliferation of precursors and the execution of effector functions. Upon antigen clearance, the CD8**^+^** T cell population contracts due to apoptosis, leaving behind a small population of memory-committed T cells (7).

T cell fate decisions are influenced by the strength and duration of antigen stimulation, costimulation, and an inflammatory signal provided by cytokines such as type I IFNs, IL-12, and IL-2 (8, 9). Availability of IL-7 and IL-15 is crucial for maintaining the pool of memory-committed progeny of CTLs (10).

The estrogen-tunable gene *Ebag*9 downregulates the killing activity of CTLs (11, 12). Deletion of *Ebag*9 in mice facilitated an enhanced release of the lytic granule marker granzyme A from CTLs. Mechanistically, EBAG9 inhibits γ2-adaptin and associates with 2 subunits of biogenesis of lysosome-related organelles complex 1, indicating that the endosomal-lysosomal trafficking route of the cytotoxic effectors is controlled by EBAG9. Consequently, in *Ebag9*^–/–^ CTLs, a profound advantage in allogeneic and tumor target cell lysis was obtained, in vitro and in vivo. In addition, clearance of *Listeria monocytogenes* infection was improved and secondary immune responses against tumor cells expressing SV-40 large T antigen (TAg) were stronger. Defects in other immune cell subsets were not detected (11).

The enhanced cytolytic efficacy mediated by *Ebag9* deletion in the primary immune response prompted us to ask whether cytolytic strength also affected memory commitment of CD8**^+^** T cells. The primary immune response is defined by the conditions for CD8**^+^** T cell activation, clonal selection, expansion, and, ultimately, a transition from an effector to a memory phase. Cytolytic mediators such as perforin and granzymes are indispensable for the effector phase because they are responsible for antigen removal and restoration of T cell homeostasis (13).

Although priming of T cells in autochthonous tumors can occur, immune responses are often insufficient in controlling tumor progression (14). This has been attributed to immune-evasive mechanisms, including the deregulation of immune checkpoints. Activation of the inhibitory pathways mediated by cytotoxic T lymphocyte–associated protein 4, programmed cell death protein 1 (PD-1), and lymphocyte activation gene-3 (LAG-3) can lead to a functional exhaustion status originally described for CD8**^+^** T cells arising during chronic infections (15). Advanced cancers exhibit overwhelming antigen exposure and an inflammatory milieu, whereas in early or premalignant lesions, neoantigens are expressed and presented to the immune system in a noninflammatory context (14, 16). Hence, the lack of an inflammatory environment could putatively shape distinct CD8**^+^** T cell priming and activation conditions.

Here, we used the *Ebag9*^–/–^ model to dissect the influence of cytolytic efficacy on CD8**^+^** T cell fate decisions, especially when mice were challenged with noninfectious stimuli. Our results revealed that EBAG9 defines threshold levels for granzyme A release and thereby contributes to the cytolytic capacity of CD8**^+^** T cells. A subsequent increase in the memory population was unrelated to the size of the phenotypic, naive precursor population or to the avidity of effector CD8**^+^** cells. We also used an autochthonous tumor model, the *Eμ-Tcl1* transgenic mouse strain in which mice develop a B cell chronic lymphocytic leukemia (CLL) that phenocopies several aspects of the human disease (17, 18). Double-transgenic *Eμ-Tcl1* *Ebag9*^–/–^ mice developed a strongly delayed leukemia progression after age 6 months, correlating with a distinct T cell activation status.

Using single-cell RNA sequencing (scRNA-Seq) of TAg oncogene-specific CD8^+^ T cells at day 7 of the effector phase, we revealed the role of transcription factors and the apoptotic pathway in the specification of larger clusters of memory-precursor Ebag9^–/–^ CD8^+^ T cells. Our model implies that cytolytic activity controls access to antigen over time and, thus, defines preferential memory T cell lineage decisions. Collectively, EBAG9 links the efficacy of the effector phase to memory programming under noninflammatory conditions.

## Results

### Ebag9^–/–^ mice develop higher proportions of HY-specific memory CD8^+^ T cells.

**Figure 1:**
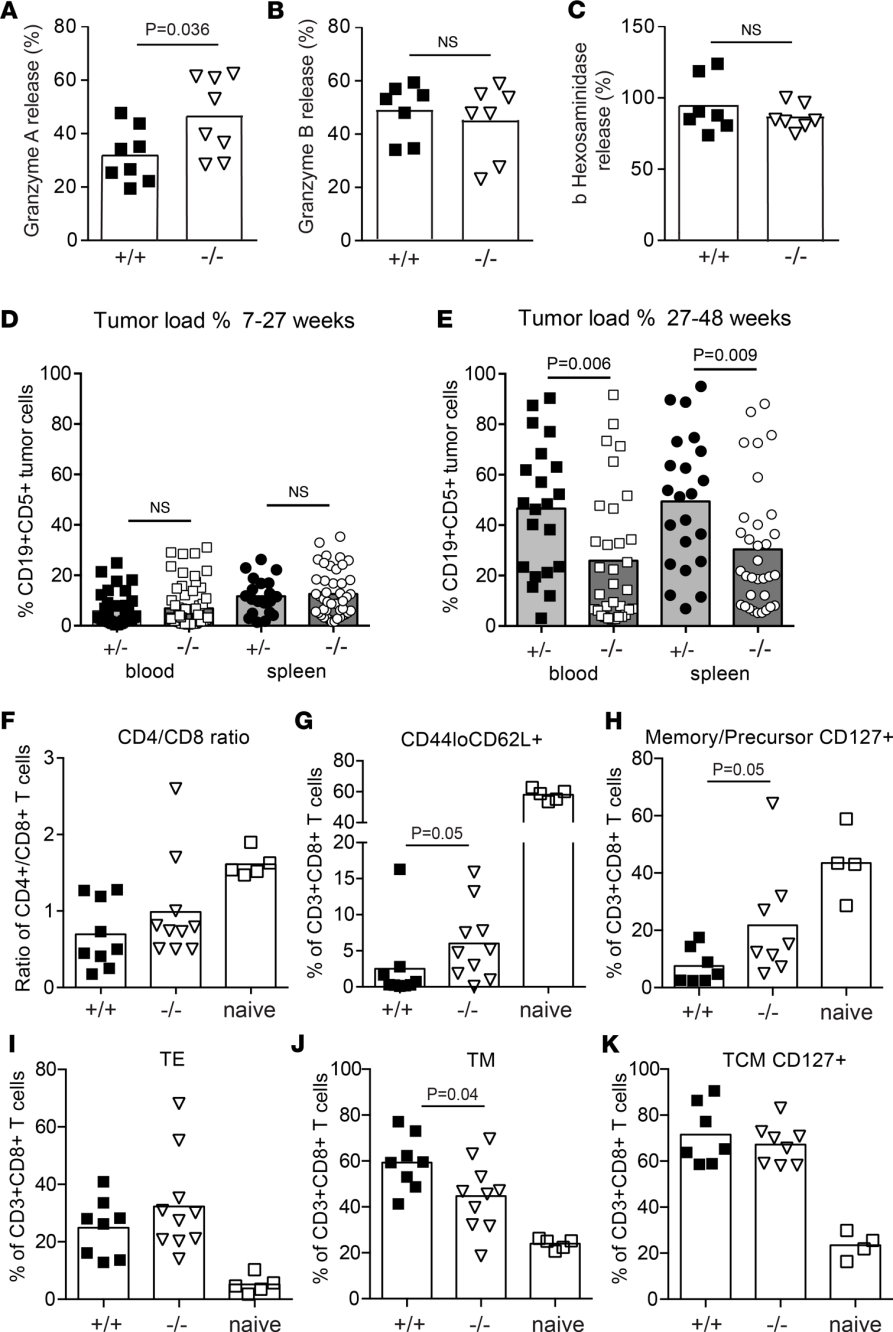
Extended control of an autochthonous tumor in Ebag9-deficient mice. (**A**) CTLs from WT (+/+) and Ebag9^–/–^ (–/–; KO) mice were generated through CD3/CD28 plate-bound stimulation. (**A**–**C**) Secretion of effector molecules was induced with anti–CD3/CD28 for 4 hours. Cell supernatant was analyzed for (**A**) granzyme A, (**B**) granzyme B, and (**C**) β-hexosaminidase enzymatic activity by colorimetric assays. Bars show the mean values ± SEM of 7–8 independent experiments. Pooled CTLs from 2–3 mice per genotype were used per assay. *P* values were determined by Mann-Whitney test. (**D** and **E**) Tumor load in spleen and peripheral blood (PB) of (**D**) 7- to 27-week-old and (**E**) 27- to 48-week-old *Eμ-Tcl1 Ebag9*^+/–^ (+/–; *n* = 21 PB; *n* = 22 spleen) and *Eμ-Tcl1 Ebag9*^–/–^ littermate mice (–/–; *n* = 33 PB; *n* = 33 spleen). CD19^+^CD5^+^ tumor cells are presented as percentages of gated lymphocytes with means. *P* values were determined by unpaired Student’s *t* test. (**F**) Cryopreserved CLL-bearing spleens were matched for age (between 6 and 10 months) and tumor load, ranging from 40% to 60% of all gated 7-aminoactinomycin D–negative lymphocytes and stained for the markers indicated. (**F**) CD4^+^/CD8^+^ ratios among CD3^+^ T cells and calculated for C57BL/B6 naive control, *Eμ-Tcl1 Ebag9*^+/+^ (+/+), and *Eμ-Tcl1 Ebag9*^–/–^ (–/–) mice. (**G**–**K**) Bar graphs report data for (**G**) CD3^+^CD8^+^CD44^lo^CD62L^+^ antigen-naive T cells; (**H**) a memory precursor subset, CD3^+^CD8^+^CD44^lo^CD127^+^; (**I**) antigen-experienced CD3^+^CD8^+^CD44^hi^CD62L^–^ effector cells (TE); (**J**) memory T cells (TM) CD3^+^CD8^+^CD44^hi^CD62L^+^; and (**K**) CD3^+^CD8^+^CD44^hi^CD127^+^ TCMs. Five independent experiments were performed with 5 naive controls, 8–10 *Eμ-Tcl1 Ebag9*^+/+^, and 10 *Eμ-Tcl1 Ebag9*^–/–^ mice. For calculation of significance, a Mann-Whitney test was used. Scatterplots show single animals; boxes and triangles represent individual animals.

*Ebag9* deletion in mice amplifies the secretion of cytolytic enzymes, leading to a profound advantage in cytotoxic T cell responses against the viral TAg antigen (11). Here, we revisited the secretory capacity of CTLs from *Ebag9*^–/–^ (KO) and WT mice by enzymatic activity assays. Induced secretion of granzyme A from *Ebag9*^–/–^ CTLs (mean ± SEM, 31% ± 10.1%) was 1.5- to 2-fold higher than from WT CTLs (mean ± SEM, 46.4% ± 14.7%) (Figure 1A). In contrast, granzyme B and β-hexosaminidase activity in the supernatants was comparable, indicating that EBAG9 influences the release of granzyme A only (Figure 1, B and C).

Next, to avoid the inherent flaws of transplantable tumors (12), we examined the functional consequences of *Ebag9*-governed cytolytic strength in an autochthonous tumor model. We monitored the spontaneous CLL development in *Eμ-Tcl1 Ebag9*^+/–^ and *Eμ-Tcl1 Ebag9*^–/–^ littermate mice. From our experiences with *Ebag9*-deleted mice, we knew that heterozygous mice behaved phenotypically exactly as *Ebag9*^+/+^ WT mice; a dosage effect due to heterozygosity at the *Ebag9* locus was not evident (11). Leukemia B cells were detected in the spleen and peripheral blood of both genotypes after more than 7 weeks, but a difference in disease onset was not obtained. At more than 6 months, *Eμ-Tcl1 Ebag9*^+/–^ mice had a further increased tumor load in spleen (for *Eμ-Tcl1 Ebag9*^+/–^, mean ± SEM, 49.4% ± 5.6%; for *Eμ-Tcl1 Ebag9*^–/–^, 30.3% ± 4.3%) and peripheral blood (for *Eμ-Tcl1* *Ebag9*^+/–^, mean ± SEM, 46.6% ± 5.6%; for *Eμ-Tcl1* *Ebag9*^–/–^, 25.9% ± 4.5%), whereas tumor load in *Eμ-Tcl1 Ebag9*^–/–^ mice was substantially lower (Figure 1, D and E, and Supplemental Figure 1A). Data showed that T cell phenotypes in *Eμ-Tcl1* mice phenocopy T cell functional hyporesponsiveness in human CLL (19). Compared with spleens from control mice (naive C57BL/6, B6), spleens of *Eμ-Tcl1* *Ebag9*^+/+^ mice exhibited a loss of CD4^+^ and a relative increase of CD8^+^ T cells (CD4^+^/CD8^+^ ratio, mean ± SEM, for control B6 spleens, 1.6 ± 0.07; for *Eμ-Tcl1* *Ebag9*^+/+^ spleens, 0.69 ± 0.35; for *Eμ-Tcl1* *Ebag9*^–/–^ spleens, 0.99 ± 0.66) (Figure 1F) and an increase in antigen-experienced CD3^+^CD8^+^CD44^hi^ cells (Figure 1I). Tumor-bearing mice acquired a stronger expansion of CD3^+^CD8^+^CD44^hi^CD62L**^+^** memory cells (median: control, 25%; *Eμ-Tcl1* *Ebag9*^+/+^, 59.4%) (Figure 1J) and a substantial loss of naive CD3^+^CD8^+^CD44^lo^CD62L^+^ T cells (median: control, 58.8%; *Eμ-Tcl1* *Ebag9*^+/+^, 0.3%) (Figure 1G and Supplemental Figure 1B).

When comparing age-matched (6–10 months) *Eμ-Tcl1 Ebag9*^–/–^ and *Eμ-Tcl1 Ebag9*^+/+^ mice, *Eμ-Tcl1 Ebag9*^–/–^ mice retained 10-fold more CD44^lo^CD62L^+^ naive CD8^+^ T cells (median: *Eμ-Tcl1 Ebag9*^+/+^, 0.31%; *Eμ-Tcl1 Ebag9*^–/–^, 4.9%) (Figure 1G) and also 3-fold more memory precursor CD8^+^CD44^lo^CD127^+^ T cells (median: *Eμ-Tcl1 Ebag9*^+/+^, 4.6%; *Eμ-Tcl1 Ebag9*^–/–^, 13.7%) (Figure 1H). Within the antigen-experienced CD8^+^ compartment, memory CD8^+^CD44^hi^CD62L^+^ T cells were significantly less frequent in *Eμ-Tcl1 Ebag9*^–/–^ animals (median: *Eμ-Tcl1 Ebag9*^+/+^, 59.4%; *Eμ-Tcl1 Ebag9*^–/–^, 46.%) (Figure 1J). No difference was obtained for T central memory cells (Figure 1K). Notably, expression of exhaustion markers LAG-3 (geometric MFI mean ± SEM: *Eμ-Tcl1 Ebag9*^+/+^: 66.9 ± 12.4 SEM; *Eμ-Tcl1 Ebag9*^–/–^: 63.7 ± 12.5 SEM) and PD-1 (geometric MFI mean *Eμ-Tcl1 Ebag9*^+/+^: 97.4 ± 17.3 SEM; *Eμ-Tcl1 Ebag9*^–/–^: 95.8 ± 15.4 SEM) within the CD3^+^CD8^+^CD44^hi^ compartment could not be discriminated between the genotypes and was only modestly higher than in naive B6 mice (Supplemental Tables 1 and 2; supplemental material available online with this article; https://doi.org/10.1172/jci.insight.155534DS1). Furthermore, CD8^+^ T cells from *Eμ-Tcl1 Ebag9*^+/+^ and *Eμ-Tcl1 Ebag9*^–/–^ mice could produce IFN-γ and TNF-α in the same amounts (Supplemental Figure 2, A–C). Collectively, *Ebag9*-deficient Tcl1 transgenic mice exhibited a distinct T cell differentiation pattern correlating with a more efficient long-term anti-CLL immune response.

In the absence of a tumor-specific antigen that would allow us to follow the T cell response against Tcl1**^+^** CLL over time, we set up an alternative antigen-specific system to examine T cell differentiation. Notably, antigen stimulation is chronic in CLL (20), whereas in our experimental model system, we used single immunization schedules. We chose the HY minor histocompatibility antigens (miHAg) encoded by genes on the Y chromosome. Clinically, anti-miHAg alloresponses after donor lymphocyte infusions contribute to graft-versus-leukemia effects (21).

**Figure 2:**
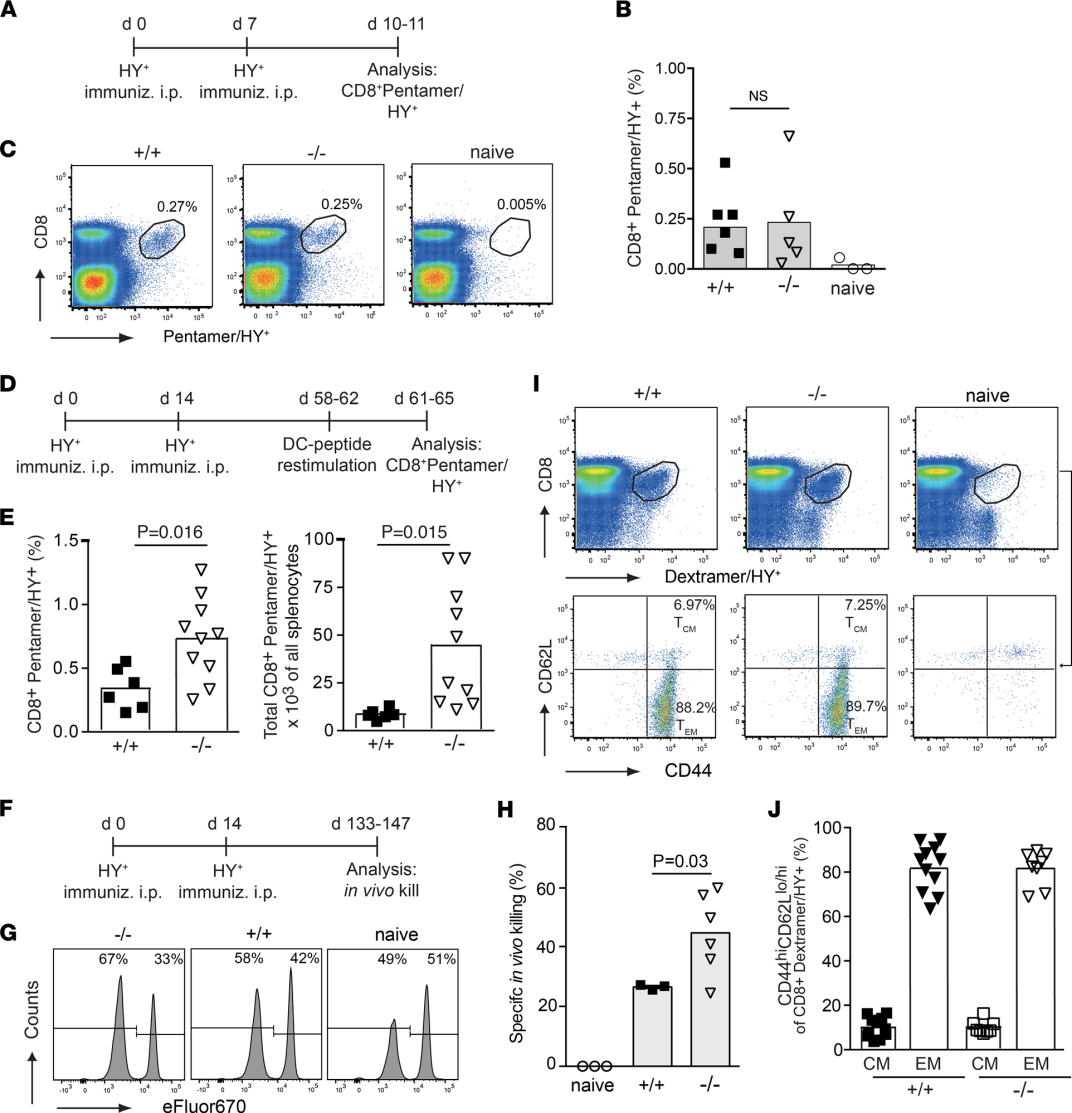
Ebag9^–/–^ mice develop a larger pool of HY-reactive memory CD8^+^ T cells. (**A**) Female WT (B6; +/+) and Ebag9^–/–^ (–/–) mice were immunized (immuniz.) twice i.p. with 5 × 10^6^ male splenocytes (HY^+^). (**B**) Splenic HY-specific CD8^+^ T cells were measured at day 10 or 11 by anti-CD8 and D^b^ Uty Pentamer staining. (**C**) Representative FACS plots are shown; gated populations represent percentages of Pentamer/HY-positive cells within the CD8^+^ population. Bars indicate mean values ± SEM; *n* = 3 independent experiments with WT (*n* = 7), KO (*n* = 5), and naive (*n* = 3) animals. A Mann-Whitney test was applied. (**D**) Immunization scheme. At days 44–48 after the last immunization, CD8^+^ T cells were restimulated in vitro for 3 days with HY-peptide–pulsed DCs. (**E**) Mean frequency of the CD8^+^/HY^+^ Pentamer of CD8^+^ T cells; (*right*) absolute numbers of all splenocytes; unpaired Student’s *t* test of 3 independent experiments with WT (*n* = 11) and KO (*n* = 8) mice. (**F**) Immunization scheme. At days 133–147 after the first immunization, mice were challenged with female and male splenocytes (1:1) labeled with different amounts of eFluor-670. The ratio of both populations was determined by flow cytometry 22 hours later. (**G**) Histograms show representative examples per group. (**H**) Specific in vivo killing reported as percentages. Bars represent mean ± SEM of 2 experiments with naive (*n* = 3), WT (*n* = 3), and Ebag9^–/–^ (*n* = 5) mice per group. A Mann-Whitney test was used for the analysis. (**I**) Mice were immunized as in **D**; HY-specific CD8^+^ T cells were detected with D^b^/HY Dextramers, without DC-HY peptide restimulation; *n* = 3 independent experiments. Representative dot plots of CD8^+^ Dextramer/HY^+^ CD44^hi^ T_CM_ and T_EM_ cells. Numbers in the gates are the percentages of T_CM_ and T_EM_ cells. (**J**) Frequency as percentages of T_EM_ and T_CM_ among all CD8^+^CD44^hi^ HY-specific T cells. Student’s *t* test was applied; each dot represents data from 1 mouse (WT, *n* = 11; KO, *n* = 8).

Female WT and *Ebag9*^–/–^ animals were immunized with male splenocytes (Figure 2A). We were unable to detect in vivo anti-HY CTL responsiveness at day 7, when the effector phase against strong infection or tumor-associated antigens usually peaks (22). Instead, at day 10 or 11, we analyzed the frequency of CD8**^+^** T cells reactive against the D^b^-restricted peptide WMHHNMDLI, which is immunodominant among the HY antigens (23). Using Pentamer/HY staining, an antigen-specific CD8**^+^** population was clearly present as a distinct population (Figure 2, B and C), but the proportion of Pentamer/HY**^+^** CD8**^+^** T cells among all splenic CD8**^+^** T cells was similar in WT (mean ± SEM, 0.20% ± 0.06%) and *Ebag9*^–/–^ (mean ± SEM, 0.23% ± 0.11%) mice. Total numbers of splenocytes and the T cell subsets do not differ between the genotypes (11).

Analysis of HY-specific CD8^+^ T cell populations, expressly antigen-experienced (CD44), terminal effector (KLRG1), or memory precursor (CD127) subsets, did not reveal a skewed phenotype between WT and Ebag9^–/–^ CD8^+^ T cells at this stage (Supplemental Figure 3, A and B) (22, 24). Thus, Ebag9 controlled the regulated release of granzyme A but did not change the numbers and proportions of primed HY-specific CD8^+^ T cells in the effector phase.

Next, we used *Ebag9*^–/–^ mice to answer the question of whether cytolytic efficacy affected the extent of CD8**^+^** memory formation. In addition, immunization with miHAg-mismatched splenocytes curtailed the inflammatory stimulus, as compared with infection models. Female WT and *Ebag9*^–/–^ mice were immunized with male splenocytes (Figure 2D), and animals were sacrificed 44–48 days after the second immunization. Isolated splenic CD8^+^ T cells were restimulated with HY-peptide pulsed DCs for 3 days. *Ebag9*^–/–^ mice developed 2-fold greater frequencies of CD8^+^/Pentamer/HY^+^–reactive T cells among all CD8^+^ T cells (mean ± SEM: WT, 0.34% ± 0.06%; KO, 0.73% ± 0.1% SEM). In accordance, total numbers in spleen were also significantly higher in *Ebag9*^–/–^ compared with WT mice (mean ± SEM: WT, 8.600 ± 1.100; KO, 44.600 ± 9.960) (Figure 2E). Besides the quantitative effect of Ebag9 deficiency on the memory T cell pool, functionally, *Ebag9*^–/–^ mice had a nearly 2-fold greater, antigen-specific, in vivo killing capacity at the memory stage compared with WT mice (mean ± SEM: WT, 26.5 ± 0.48; KO, 44.5 ± 5.5) (Figure 2, F–H).

Commitment to the T central memory (T_CM_) versus T effector memory (T_EM_) lineage occurs during the primary response. Weak signaling induced by low availability of antigen-presenting cells (APCs) or high numbers of T cell precursors favors T_CM_ (CD8**^+^**/Dextramer/HY**^+^**/CD44^hi^/CD62L**^+^**) generation. In contrast, increasing the ratio of APCs to CD8^+^ T cells preferentially promotes T_EM_ (CD8**^+^**/Dextramer/HY**^+^**/CD44^hi^/CD62L^–^) development (25). At days 43–47 after the second immunization, both genotypes developed similar ratios of T_EM_ and T_CM_ subsets among their HY-specific CD8 memory population (mean ± SEM for WT: T_EM_, 81.7% ± 3.2%, T_CM_, 10.05% ± 1.4%; for KO: T_EM_, 81.6% ± 2.8%, T_CM_, 10.18% ± 1.05%) (Figure 2, I and J). Thus, similar ratios of the T_CM_ and T_EM_ lineages would be consistent with identical precursor numbers during priming.

Because differences in the frequencies of antigen-specific, naive T cell precursors (CD8**^+^**CD62L^hi^) might be responsible for the divergent anti-HY memory formation, we examined the TCR repertoire under homeostatic naive conditions. Using a TCR β chain–specific staining of CD8**^+^**CD62L^hi^ cells, repertoire perturbations in female *Ebag9*^–/–^ animals, compared with B6 WT mice, were not seen (Supplemental Figure 3C). Next, we also examined the TCR repertoire in an antigen-experienced CD8**^+^** population. At days 48–52 after immunization, splenic CD8^+^ T cells were purified and co-cultured with HY-peptide–pulsed DCs for 3 days. Viable CD8^+^CD44^hi^ T cells were co-stained with a Vβ-specific Ab panel, but significant differences in the frequencies of Vβ usage were not revealed (Supplemental Figure 3D). Therefore, it seemed unlikely that naive CD8^+^ T cells from Ebag9^–/–^ mice had a selective advantage caused by an altered antigen-specific TCR or a differential clonal expansion.

To analyze the possibility that the increased amount of *Ebag9*^–/–^ memory CD8^+^ T cells is based on a survival advantage of effector cells, we analyzed HY-specific CD8^+^ T cells from WT and *Ebag9*^–/–^ mice by annexin V staining. Despite some variances within the WT group, no significant differences were observed between the 2 genotypes (Supplemental Figure 3, E and F). In addition, the proliferation rate was examined. A mixed lymphocyte reaction with irradiated, male stimulator splenocytes and female responders was performed. CD8**^+^** T cells were used in an MTT assay, which is broadly used as an indicator of metabolic activity and proliferation (26). Both genotypes exhibited the same substrate conversion rate and, thus, grew similarly (Supplemental Figure 3G).

To further examine T cell fitness, FACS-sorted HY-specific CD8^+^ T cells from WT and *Ebag9*^–/–^ mice at day 11 after the first immunization were analyzed for gene expression by reverse transcription quantitative PCR (Supplemental Figure 3H). For the anti-apoptotic and memory cell–promoting gene *Bcl-2*, a statistically relevant difference between WT and Ebag9-deficient CD8^+^ T cells was not obtained. Likewise, a significantly differential expression was not observed for *Tcf7* that encodes for the CD8^+^ memory T cell–promoting transcription factor Tcf1. Instead, *Id3*, coding for the inhibitor of DNA binding 3, was significantly upregulated 1.6-fold (*P* = 0.049), whereas the counteracting transcription factor Id2 remained unchanged. Both factors act in pairs, and *Id*3 supports the generation of long-lived memory CD8^+^ T cells.

Regulatory T cells could be required to counterbalance the increased CD8^+^ effector capacity in *Ebag9*^–/–^ mice (27). Under homeostatic conditions, *Ebag9*^–/–^ mice exhibited no alteration of the CD4**^+^**CD25**^+^** and CD4**^+^**FoxP3**^+^** population in secondary lymphoid organs, thymus, or blood (Supplemental Figure 3, I and J). Taken together, *Ebag9* deletion conferred on mice a selective advantage for the development of a larger miHAg-specific CD8**^+^** memory pool.

### Enhanced memory formation of Ebag9^–/–^ CD8^+^ T cells in a mixed-chimera model.

**Figure 3:**
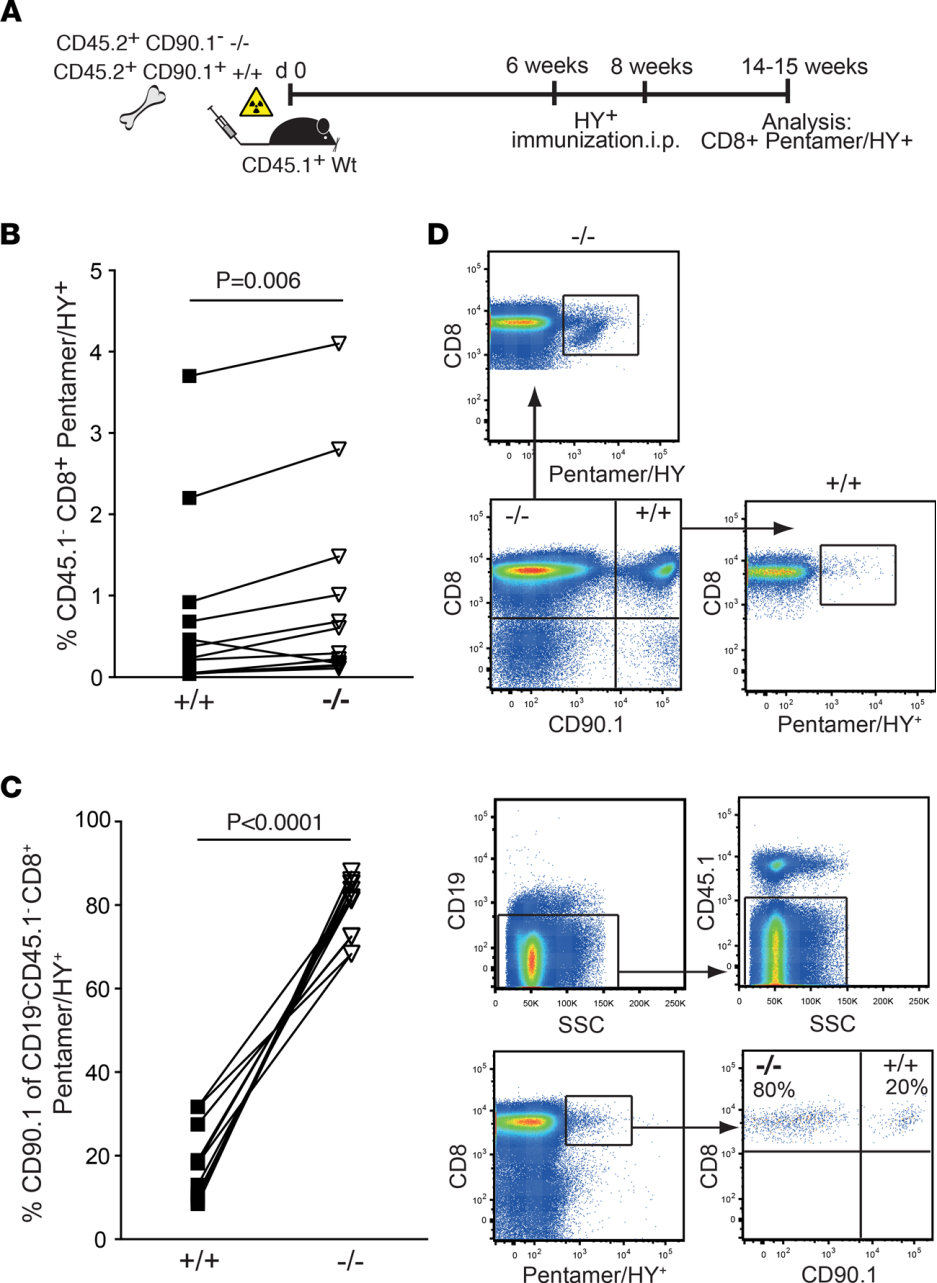
CD8^+^ T cell memory formation in Ebag9^–/–^ mice during noninflammatory priming. (**A**) CD45.1^+^Thy1.2^+^ congenic recipient mice were lethally irradiated and transplanted with a 1:1 mixture of 2.5 × 10^6^ CD45.2^+^CD90.1^+^ (WT, Ebag9^+/+^) and 2.5 × 10^6^ CD45.2^+^CD90.1^–^ (Ebag9^–/–^) BM cells. After 6 and 8 weeks, chimeric mice were immunized i.p. with male (HY^+^) splenocytes. At 44–48 days after the last immunization, splenic CD8^+^ T cells were MACS bead–enriched and analyzed without further re-stimulation. (**B**) Differentiation of congenic donor WT (+/+) and *Ebag9*^–/–^ (–/–) CD8^+^ T cells by anti-CD90.1 staining; residual CD45.1^+^ host cells were excluded. Analysis of HY-specific CD8^+^ T cells using Pentamer/HY staining; *n* = 3 independent experiments; WT mice, *n* = 13; KO mice, *n* = 13. The graph depicts the frequencies in percentages of HY-reactive CD8^+^ T cells among the CD90.1^–^ (*Ebag9*^+/+^) and CD90.1^–^ (WT) CD8^+^ T cell population in single recipient mice (CD45.1^+^CD90.1^–^). (**C**) Ebag9-deficient HY-reactive T cells predominate over the congenic WT response. The ratios (%) of Pentamer/HY-specific CD8^+^ T cells derived from either WT (CD19^–^CD45.1^–^CD8^+^ Pentamer/HY^+^ CD90.1^+^) or Ebag9^–/–^ (CD19^–^CD45.1^–^CD8^+^ Pentamer/HY^+^ CD90.2^+^) progenies are given for each chimeric recipient mouse (*n* = 13 mice per genotype). SSC, side scatter. (**B** and **C**) A paired *t* test was applied. (**D**) A representative FACS dot plot for the detection of CD8^+^/Pentamer/HY^+^ T cells within the gated congenic CD90.1^+^ and CD90.1^–^ splenic population is shown. Host cells were excluded by gating on CD45.1^–^ transferred CD8^+^ T lymphocytes.

To interrogate the influence of microenvironmental factors on memory formation in *Ebag9*^–/–^ mice, we generated mixed BM chimeras. Lethally irradiated, congenic CD45.1**^+^**CD90.1^–^ female mice were transplanted with equal numbers of female WT-derived CD45.2**^+^**CD90.1**^+^** and *Ebag9*^–/–^-derived CD45.2**^+^**CD90.1^–^ BM cells. After hematopoietic reconstitution, mice were immunized with male splenocytes and analyzed 6–7 weeks later (Figure 3A). Between individual responders, the frequencies of Ebag9^–/–^ and WT Pentamer/HY**^+^** CD8^+^ T cells varied to some extent. However, congenic CD90.1^–^CD8**^+^**/Pentamer/HY**^+^** progeny that originated from *Ebag9*^–/–^ donors (mean ± SEM: for KO, 0.92% ± 1.2%; for WT, 0.69 ± 1.0) were substantially more frequent among all CD8**^+^** cells and expanded to larger ratios, compared with WT progeny (CD90.1**^+^**CD8**^+^**/Pentamer/HY**^+^**) (Figure 3, B–D) (mean ± SEM: for WT, 18.15% ± 8.7%; for KO, 79.6% ± 6.8%).

To exclude that the predominance of *Ebag9*^–/–^ T cells in the memory T cell population was driven by a better engraftment of *Ebag9*^–/–^ BM, a nonimmunized control group was analyzed (Supplemental Figure 4). Six weeks after transferring equal numbers of WT-derived CD45.2^+^ Ubc-GFP^+^ and *Ebag9*^–/–^-derived CD45.2^+^ Ubc-GFP^–^ BM cells into lethally irradiated CD45.1^+^ recipient mice, an equal distribution of GFP^+^ WT and GFP^–^ *Ebag9*^–/–^ cells was observed (Supplemental Figure 4, A–D). Confirming the results from the CD90.1 congenic system (Figure 3A), in response to immunization, antigen-specific CD8^+^CD44^+^ memory T cells originated at higher frequencies from *Ebag9*^–/–^ donors (Supplemental Figure 4, E–H).

Collectively, these experiments suggested that when challenged with identical amounts of antigen, deletion of Ebag9 favored a stronger antigen-specific memory CD8**^+^** T cell formation.

### Memory formation of monoclonal MataHari TCR transgenic T cells resembles the differentiation process in the polyclonal anti-HY T cell population.

**Figure 4:**
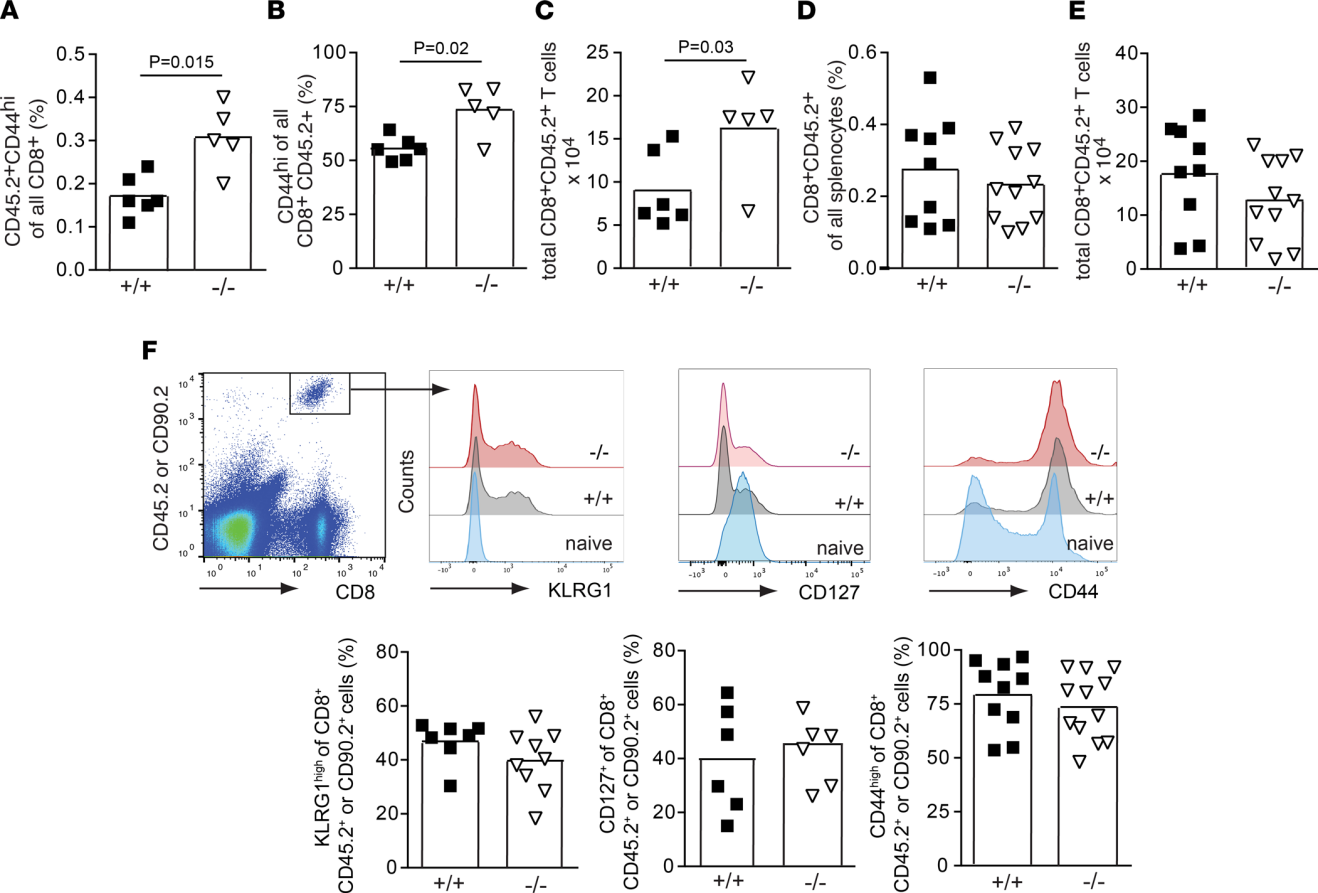
CD8^+^ T cells derived from Ebag9-deficient MataHari mice develop a stronger HY-specific memory response. (**A**) Female mice (CD45.1^+^) were transferred (i.v.) with 4× 10^4^ to 5 × 10^4^ sorted CD8^+^TCR Vβ 8.3^+^ CD62L^hi^ T lymphocytes derived from *Ebag9*^+/+^ × MataHari (+/+) or *Ebag9*^–/–^ × MataHari (–/–) female donors (CD45.2^+^). One day later, recipients were immunized i.p. with 5 × 10^6^ male splenocytes. The frequency of CD8^+^CD45.2^+^CD44^hi^ T cells as percentages of all CD8^+^ T cells was determined >50–56 days after immunization, without in vitro restimulation. (**B**) The percentage of CD44^hi^ T cells among all CD45.2^+^CD8^+^ T cells is reported. (**C**) Total numbers of CD8^+^CD45.2^+^CD44^hi^ memory T cells in spleen are depicted. Each dot represents data from 1 congenic recipient mouse (CD45.1). WT mice, *n* = 6; KO mice, *n* = 5. Bars indicate mean percentage values; *n* = 2 independent experiments. A Mann-Whitney test was used for this analysis. (**D**) As in **A**, congenic female mice were transferred with CD8^+^TCR Vβ 8.3^+^ CD62L^hi^ T lymphocytes sorted from *Ebag9*^+/+^ × MataHari (+/+) or *Ebag9*^–/–^ × MataHari (–/–) female donors (CD45.2^+^). Animals were immunized as before, and splenic MataHari cells were detected by the expression of CD8^+^CD45.2^+^ at day 7. Graph presents the frequencies of CD8^+^CD45.2^+^ cells among all splenocytes; the bars indicate mean values. (**E**) Total numbers of this population; *n* = 3 independent experiments. WT mice, *n* = 9; KO mice, *n* = 11. (**D** and **E**) Student’s *t* test was used. (**F**) *Top*: A representative dot plot shows the gating strategy for detection of MataHari T cells. In some cases, recipient mice were congenic for CD90.1^+^. Differentiation of CD45.2^+^CD8^+^ or CD90.2^+^CD8^+^ population was further analyzed by anti-CD44, anti-KLRG1, and anti-CD127 staining. Overlay histograms show shifts relative to CD8^+^ naive T cells. *Bottom*: Bar charts report quantitation of marker expression. Bars show mean values; *n* = 3 independent experiments with 7–10 WT and 6–12 KO animals. Student’s *t* test was applied.

To exclude potential alterations in TCR repertoire, affinities, and precursor frequencies, we crossed *Ebag9*^–/–^ mice with MataHari transgenic mice whose TCR recognizes the HY-epitope (28). Of note, as shown in Figure 2D, we assessed the anti-HY response in an endogenous repertoire, and therefore, we used a different time scale for immunization and analysis. We adoptively transferred 4 × 10**^4^** 5 × 10**^4^** female, naive (CD8**^+^**CD62L^hi^) WT and *Ebag9*^–/–^ MataHari CD8**^+^** T cells (CD45.2**^+^**Vβ8.3^+^) into CD45.1 female recipients and immunized them with male splenocytes (Supplemental Figure 5). At 50–56 days postimmunization, we quantified splenic CD8^+^CD45.2^+^CD44^hi^ memory T cells. To exclude the effects of a secondary immune response, frequencies were determined without additional in vitro restimulation (Figure 2, D and E). *Ebag9*^–/–^-derived MataHari CD8^+^CD45.2**^+^** T cells exhibited higher frequencies among all CD8^+^ splenocytes (mean ± SEM: for WT, 0.17% ± 0.01%; for KO, 0.31% ± 0.03%) (Figure 4A), but also higher ratios of CD44^hi^ memory cells within the CD8^+^CD45.2^+^ population (Figure 4B). Total numbers (Figure 4C) of CD8^+^CD45.2**^+^** T cells derived from *Ebag9*^–/–^ mice were 1.8-fold higher (mean ± SEM: for WT, 9.0 × 10^4^ ± 1.76 × 10^4^; for KO, 16.2 × 10^4^ ± 2.56 × 10^4^). Absolute numbers of splenocytes in the CD45.1**^+^** recipient mice were comparable (mean ± SEM: for WT, 5.15 × 10^7^ ± 0.5 × 10^7^; for KO, 5.16 × 10^7^ ± 0.6 × 10^7^).

These results prompted us to investigate whether lineage commitment after transfer of MataHari T cells mimicked our observations of polyclonal precursor activation. Identical numbers of WT or *Ebag9*^–/–^ MataHari T cells were transferred into CD45.1**^+^** female recipients (day 0), followed by immunization with male splenocytes. At day 7, *Ebag9*^–/–^ exhibited similar expansion as WT MataHari T cells, as indicated by the proportions of CD8**^+^**CD45.2**^+^** cells among all splenocytes (mean ± SEM: for WT, 0.27% ± 0.04%; for KO, 0.23% ± 0.03%) (Figure 4D). Likewise, total numbers of these antigen-specific T cells were comparable (mean ± SEM: for WT, 17.6 10^4^ ± 0.3 × 10^4^; for KO, 14.2 10^4^ ± 0.2 × 10^4^) (Figure 4E). Compared with WT controls, *Ebag9*^–/–^ MataHari T cells had an identical proportion of activated CD44^hi^CD8**^+^** T cells (mean ± SEM: for WT, 79.21% ± 5%; for KO, 73.53% ± 4.4%), and also similar frequencies of precursor memory CD127**^+^** (mean ± SEM: for WT, 39.73% ± 8.14%; for KO, 45.3% ± 5%) and terminal effector KLRG1^hi^CD8**^+^** T cells (mean ± SEM: for WT, 46.9% ± 2.9%; for KO, 39.7% ± 3.8%) among all congenic CD45.2**^+^** or CD90.2^+^ cells (Figure 4F).

Collectively, a surface marker–defined phenotypic distinction between terminal effector and precursor memory CD8**^+^** T cells could not resolve the early memory differentiation processes associated with *Ebag9* deletion.

### The TAg tumor neoantigen induces similar antigen-specific CD8^+^ memory differentiation as the miHAg system.

To enable a better kinetic comparison with infection models, we next analyzed the expression of differentiation markers following i.p. immunization with TAg-expressing Co16.113 tumor cells. Together with the slow tumor growth rate, this comparably low dose of tumor cells may better mimic the tumor antigen challenge at tumor onset as compared with larger numbers of transplanted tumor cells (29). Furthermore, a low dose of tumor cells may enable the analysis of CD8^+^ memory formation as a result of antigen recognition under noninflammatory conditions. Analysis of serum samples gained from mice at days 2 and 7 after immunization with TAg-expressing Co16.113 tumor cells for inflammatory cytokines revealed a noninflammatory condition: no significant differences in cytokine concentration (e.g., IL-1α, IL-6, MCP-1) between naive and immunized mice were detected (Supplemental Figure 6). Strikingly, serum concentrations of several prominent inflammatory mediators, like IFN-β, IFN-γ, TNF-α, IL-1β, and IL-17a, were even below the detection threshold of the cytokine bead assay, a result also confirmed for naive mice.

**Figure 5:**
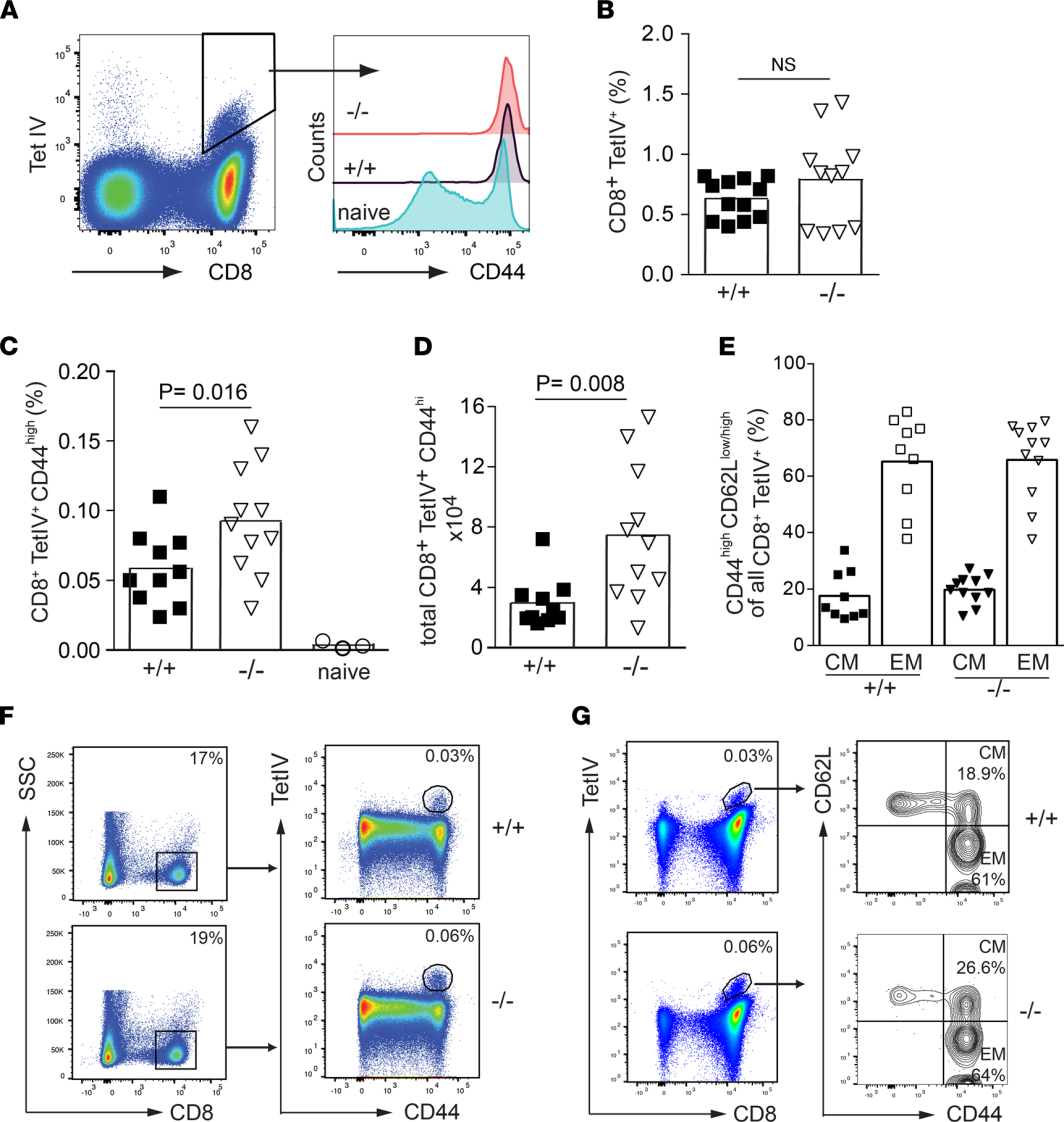
Immunization with the strong TAg-neoantigen under noninflammatory conditions induces a higher CD8^+^ T cell memory response in Ebag9^–/–^ mice. (**A**) WT (+/+) and *Ebag9*^–/–^ (–/–) mice were immunized i.p. with 1 × 10^6^ TAg^+^ 16.113 tumor cells. At day 6, splenic CD8^+^ T lymphocytes reactive to the immunodominant peptide IV antigen were detected with a K^b^/IV Tetramer (Tet IV). A representative dot plot shows an antigen-specific CD8^+^Tet IV^+^ population is gated. (*Right*) CD44 expression indicates activation; *n* = 2 independent experiments. (**B**) In the effector phase, WT and *Ebag9*^–/–^ show similar frequencies of TAg-specific CD8^+^ T cells. Frequencies of CD8^+^/Tet IV^+^ T cells among all splenocytes are shown. Student’s *t* test was applied; WT mice, *n* = 6–12; KO mice, *n* = 6–11. (**C**) Increased frequencies and numbers of Tet IV-reactive CD8^+^CD44^hi^ T cells in *Ebag9*^–/–^ mice. WT and Ebag9^–/–^ mice were immunized as in **A**; 55–65 days later, splenocytes were stained without in vitro restimulation with anti-CD8, anti-CD44, and Tet IV tetramers. Frequencies are reported as percentages of CD8^+^Tet IV^+^CD44^hi^ T cells among all splenocytes. (**D**) Total numbers of CD8^+^Tet IV^+^CD44^hi^ T cells within spleens. Bars indicate mean values; *n* = 3 independent experiments, with 10 WT and 11 KO mice. Student’s *t* test was applied. (**E**) Distinction between T_CM_ and T_EM_ cells made by CD44^+^CD62L^lo^ (EM) or CD44^+^ CD62L^hi^ (CM) co-staining. Bar graph indicates the frequency, in percentages, of T_EM_ and T_CM_ among all CD8^+^CD44^hi^ TetIV-specific T cells. (**F** and **G**) Representative dot plots show the gating strategy for TAg-specific (Tet IV) CD8^+^ T cell detection and distinction into T_CM_ and T_EM_ cells. Numbers in the gates are the percentages of antigen-specific T cells, T_CM_, and T_EM_ cells.

Beginning at day 6, we obtained a robust and distinct population of Tetramer/TetIV**^+^** CD8**^+^** T cells in spleen (Figure 5A). The frequencies of TAg-specific CD8**^+^** T cells among total splenocytes were similar in WT and *Ebag9*^–/–^ mice (mean ± SEM: for WT, 0.63% ± 0.05%; for KO, 0.78% ± 0.11%) (Figure 5B). In both genotypes, a uniform activation of the antigen-specific CD8^+^CD44^hi^ population was observed (Figure 5A).

Next, WT and *Ebag9*^–/–^ mice were immunized with Co16.113 tumor cells. After 55–65 days, the frequency of Tetramer/TetIV**^+^**CD44^hi^CD8**^+^** T cells among all splenocytes was increased in *Ebag9*^–/–^ mice (mean ± SEM: for WT, 0.058% ± 0.01%; for KO, 0.092% ± 0.01%). Correspondingly, total numbers of antigen-specific CD8**^+^** memory T cells were also elevated (mean ± SEM: for WT, 2.97 ± 0.52 × 10^4^; for KO, 7.46 ± 1.37 × 10^4^) (Figure 5, C and D). Both genotypes developed similar ratios of T_EM_ and T_CM_ subsets among their antigen-specific CD8^+^ T cell memory population (Figure 5, E–G).

### scRNA-Seq reveals distinct subpopulations and transcriptional regulation during CD8^+^ T cell memory formation in Ebag9^–/–^ mice.

**Figure 6:**
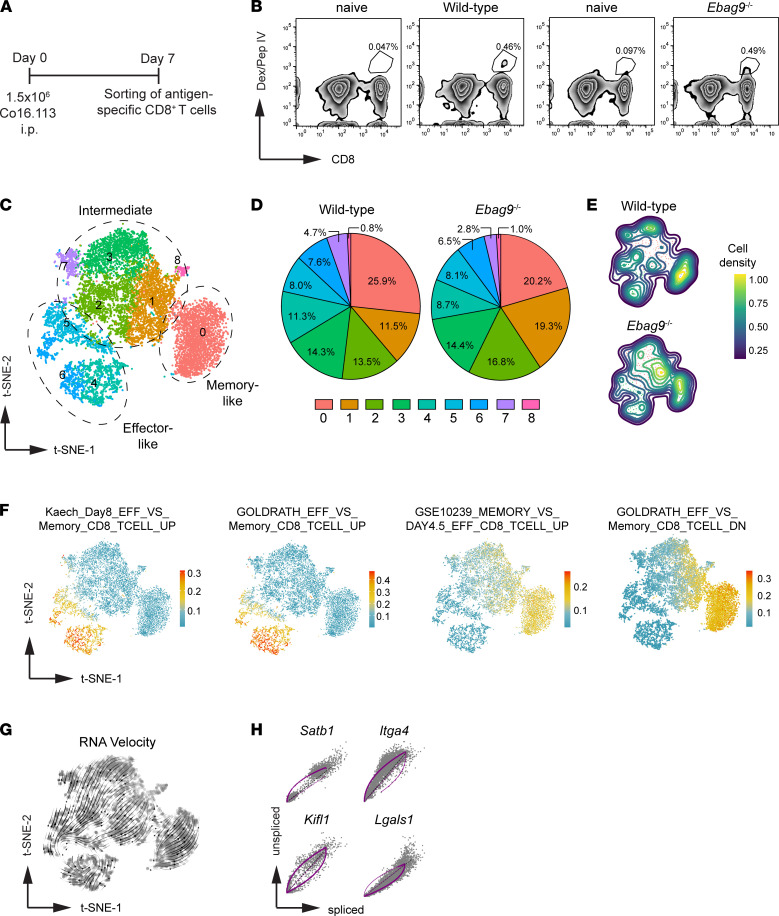
Single-cell transcriptomics reveals that TAg-specific CD8^+^ T cells from Ebag9^–/–^ mice are endowed with enhanced memory commitment. (**A**) WT and *Ebag9*^–/–^ mice were immunized (i.p.) with 1.5 × 10^6^ TAg^+^ Co16.113 tumor cells. At day 7, splenic CD8^+^ T lymphocytes were sorted with a peptide IV dextramer (Dex/Pep IV). Cells were pooled from 4–5 donors for each genotype. (**B**) Gating strategy for FACS sorting of antigen-specific CD8^+^ T cells from WT and *Ebag9*^–/–^ mice. Naive mice were used as a control. Frequencies of CD8^+^ Dex/PepIV^+^ T cells are indicated as numbers on the gate. (**C**) t-Distributed stochastic neighbor embedding (t-SNE) projection of WT (*n* = 4301 cells) and *Ebag9*^–/–^ (*n* = 6938 cells) cells based on transcriptional profiles. Each dot represents a cell. The assigned 8 different clusters of cells are presented in different colors. (**D**) Distribution of clusters across all WT and *Ebag9*^–/–^ cells examined is shown. (**E**) t-SNE embedding displays cell density distribution across all analyzed WT and *Ebag9*^–/–^ cells. (**F**) t-SNE embedding of merged WT and *Ebag9*^–/–^ profiles colored by cell-level pathway signature scores for gene sets in the C7 immunological pathway collection from the molecular signatures database. (**G**) RNA velocities of merged WT and *Ebag9*^–/–^ cells are visualized on the t-SNE projection. Arrows indicate the direction toward the likely future states. (**H**) Cell differentiation–associated genes important for T cell maturation and function with pronounced dynamic behavior (i.e., highest velocity) are depicted.

Given the inability to use surface marker staining to reveal quantitative differences in lineage-committed terminal effector and precursor memory T cells (Figure 4F and Supplemental Figure 3, A and B), we applied scRNA-Seq to compare transcriptional programs in antigen-specific CD8^+^ T cells in vivo. Mice were immunized with Co16.113 tumor cells. At day 7 postimmunization, antigen-specific CD8**^+^** Dextramer IV**^+^** T cells were isolated by FACS sorting for gene-expression analysis using scRNA-Seq (10x Genomics) (Figure 6, A and B, and Supplemental Figure 7A).

After applying the Seurat integration workflow (30) and quality control for number of genes per cell and mitochondrial gene content, 4301 WT and 6938 *Ebag9*^–/–^ TAg-specific CD8^+^ T cells were analyzed. Unsupervised clustering of antigen-specific CD8^+^ T cells assigned cells distinctly into 8 clusters on the basis of their transcriptomes (Figure 6C). In particular, a higher proportion of *Ebag9*^–/–^ cells was present in cluster 1, whereas WT cells were more frequently found in cluster 0 (Figure 6, D and E, and Supplemental Figure 7B).

The clusters could be identified as memory-like (cluster 0), effector-like (clusters 4, 5, and 6), or intermediate (clusters 1, 2, 3, 7, and 8) CD8^+^ T cells (Figure 6C and Supplemental Figure 7C). Cluster 0 cells expressed genes associated with memory T cell differentiation, such as *Il7r*, *Sell* (encoding CD62L), *Ccr7*, *Cd27*, *Lef1*, *Tcf7* (encoding TCF1), and *Zeb1* (Supplemental Figure 7E). Likewise, cluster 1 and 8 cells expressed high levels of memory or precursor memory signature genes (*Il7r*, *Cd27*, *Tcf7*, and *Id3*). *Sell* and *Ccr7* were only slightly expressed. In addition, some effector-associated genes, such as *Cxcr3*, *Id2*, and *Il2rb*, were enriched in these clusters of cells (Supplemental Figure 7, D and E). In clusters 4, 5, and 6, genes associated with effector T cell function involving *Gzma*, *Gzmb*, *Il2rb*, *Cxcr3*, and *Id2* were prominently expressed (Supplemental Figure 7D). Genes important for cell cycle control (i.e., *Cks2*, *Lig1*, *Stmn1*, and *Cdca8*), were also upregulated among those cells (Supplemental Figure 7G). Cells within clusters 2, 3, and 7 also expressed genes associated with effector T cell activity. Simultaneously, the memory-related genes *Il7r*, *Tcf7*, and *Cd27* were enriched (Supplemental Figure 7, D and E). Furthermore, exhaustion-related gene expression (*Klrg1*, *Pdcd1*, *Cd160*, *FasL*, *Cxcr6*) was detected among all cell clusters, except for memory cluster 0 (Supplemental Figure 7F).

Next, we applied gene sets defined by the immunological C7 collection from the Molecular Signature Database (31) to confirm the biologically relevant phenotypes of the defined clusters. Clusters 4, 5, and 6 were associated with gene sets previously shown to be upregulated in effector T cells. Gene sets associated with a memory T cell phenotype were preferentially associated with clusters 0, 1, and 8 (Figure 6F).

Applying RNA velocity (32), a directional flow from proliferative effector-associated CD8^+^ T cells (clusters 4, 5, and 6) through the intermediate state (clusters 1, 2, 3, 7, and 8) toward memory CD8^+^ T cells (cluster 0) (Figure 6G) was detected. Major dynamic-driving genes for the inferred T cell dynamics were the upregulated genes *Satb1*, *Itga4*, and *Kifl1* with a positive velocity, as well as the downregulated *Lgals1* gene, for which a negative velocity was detected (Figure 6H).

**Figure 7:**
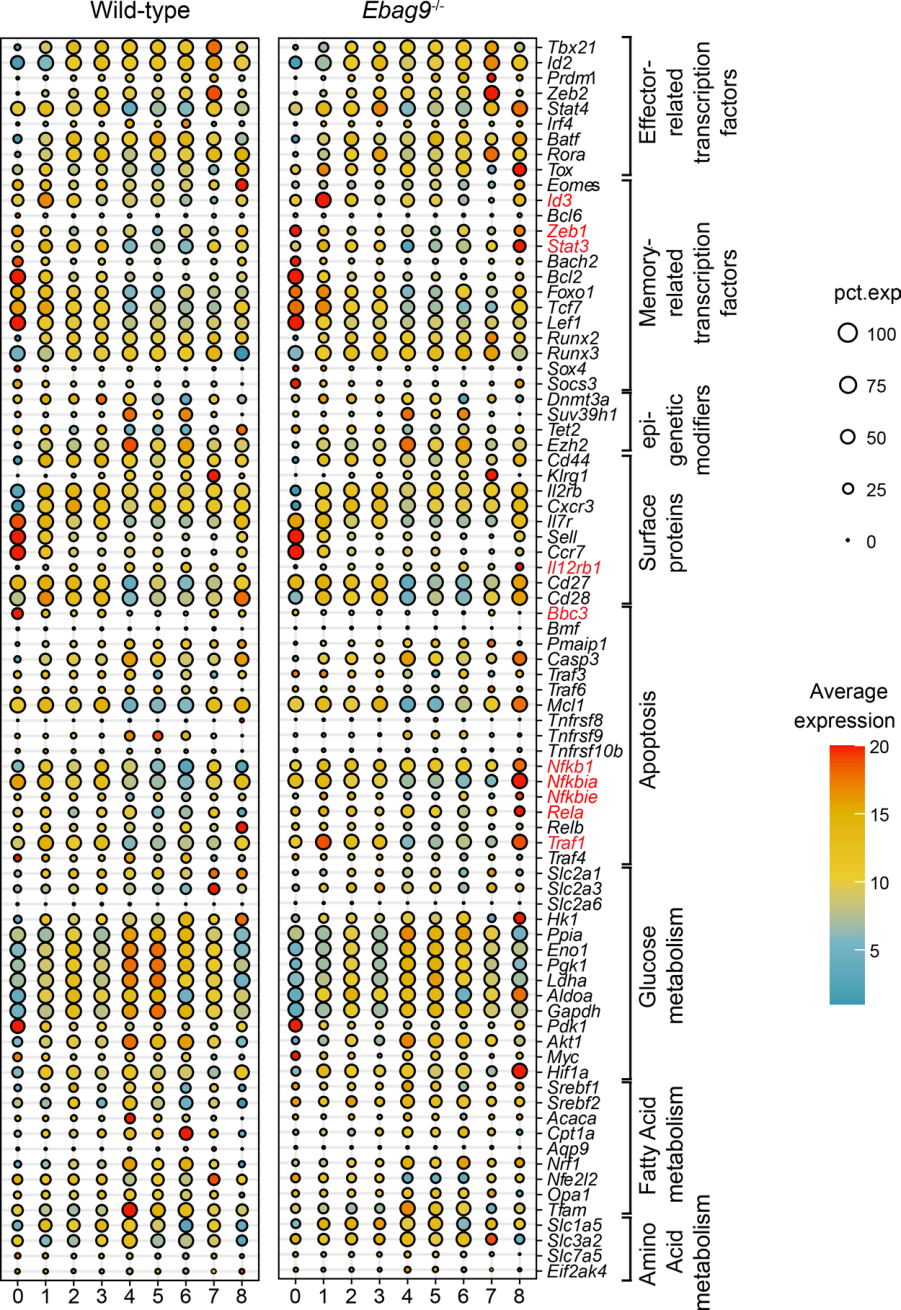
Key genes associated with memory commitment and survival are upregulated in TAg-specific CD8^+^ T cells from *Ebag9*^–/–^ mice. Dot plot representing the size-coded percentage of cells expressing selected genes involved in the differentiation of CD8^+^ T cells for WT and *Ebag9*^–/–^ cells among all clusters. Expression changes are depicted according to the color scale. Gene names with the most differential expression between WT and *Ebag9*^–/–^ cells are depicted in red. The ranges of the cell numbers are represented by the diameter of the circles; each column represents 1 cluster. pct. exp, percentage expression.

In addition, we analyzed the expression of key genes associated with either effector or memory commitment within the different clusters of WT and *Ebag9*^–/–^ cells (Figure 7). Most interestingly, a higher expression of the transcription factor *Id3* was observed for *Ebag9*^–/–^ cluster 1 cells. Furthermore, *Ebag9*^–/–^ cluster 0 cells expressed *Zeb1* and cluster 8 cells expressed *Stat3* and *I12rb1* to a higher extent than the corresponding WT cells of those clusters.

Gene expression of the strongly pro-apoptotic, BH3-only protein BBC3 was increased in memory-associated cluster 0 WT cells (Figure 7). On the contrary, *Ebag9*^–/–^ cells associated with the memory-related clusters 1 and 8 had a higher expression of *Traf1*. An inhibitory effect of this TNF receptor–associated factor 1 (TRAF1) on antigen-induced apoptosis of CD8^+^ T cells was shown in transgenic mice overexpressing TRAF1 (33). Furthermore, *Ebag9*^–/–^ cluster 8 cells revealed a higher expression of genes related to the NF-κB pathway (*Nfμb1*, *Nfμbia*, *Nfμbie*, and *Rela*) (Figure 7). Combined, their activity would be consistent with an anti-apoptotic function (34, 35).

Metabolism is an important regulator of T cell differentiation (36). Proliferative WT cells of the effector-associated clusters 4, 5, and 6 were the most metabolically active cells, exhibiting slight upregulation of most of the metabolic genes (Figure 7). This may indicate their ongoing engagement in the antigen removal during the effector phase, a reaction already dampened in the Ebag9^–/–^ T cell population.

Taken together, scRNA-Seq revealed transcriptional heterogeneity among antigen-specific CD8^+^ T cells from *Ebag9*^++^ and *Ebag9*^–/–^ mice. These T cells were distributed at differential frequencies among the clusters, with a gain in *Ebag9*^–/–^ cluster 1 defined by memory-promoting gene functions.

## Discussion

Using *Ebag9*-deleted mice allowed us to assess the consequences of an altered cytolytic strength on CD8**^+^** lineage decisions in conjunction with the absence of a strong inflammatory stimulus (6, 37). *Ebag9*^–/–^ mice immunized with miHAg-mismatched male splenocytes developed significantly higher frequencies and numbers of HY-specific CD8**^+^** T cells in the memory phase. These results suggested that the encounter with a miHAg under noninflammatory priming conditions induced a differentiation program driving CD8**^+^** T cells to gain preferentially memory properties.

To get more insight into the systemic consequences of *Ebag9* deletion in a model of chronic antigen stimulation, we used the transgenic *Eμ-Tcl1* CLL mouse model. Immune responses against transplanted tumor cells, as used in a previous study, have a greater risk of misinterpretation because they need to be injected in large numbers, usually grow fast, may have acquired phenotypic changes in vitro, and challenge the T cell response with rapidly dying cells at a single time point (12, 29). In contrast, *Eμ-Tcl1* mice phenocopy human CLL regarding an immunosuppressive phenotype characterized by aberrant T cell subsets and an impaired ability to form immunologic synapses (19, 20). Additionally, the disease proceeds rather slowly, which might favor the acquisition of secondary mutations and neoantigens. Putting our findings together, we conclude that T cells potentially respond better to that challenge depending on their activation status and subset differentiation. Using the *Eμ-Tcl1* leukemia cell transplantation model, it has been shown that PD-1 immune checkpoint blockade can restore CD8 T cell function and delay leukemia progression (38, 39). In the present study, applying an autochthonous tumor model, we found that enhanced cytolytic activity endowed *Eμ-Tcl1 Ebag9*^–/–^ mice with a better clinical outcome compared with *Eμ-Tcl1 Ebag9*^+/–^ mice, suggesting that immune escape can also be restored at the level of cytolytic capacity. T cell differentiation in *Ebag9*^–/–^ CLL mice was different from that in *Ebag9* WT mice, because significantly more naive CD8^+^ T cells and fewer antigen-experienced memory cells were found. Although there are some discrepancies between our study and previous studies in transplanted immune checkpoint blockade–treated mice, we also noted in *Ebag9*^–/–^ CLL mice trends in T cell subset repair similar to programmed cell death ligand 1 blockade (38). The *Ebag9*^–/–^ model does not exclude that other immune cell subsets also contribute to better antitumor effector functions, because the gene was not deleted in a T cell–specific manner.

Inflammation influences the magnitude of proliferative expansion as well as the rate at which antigen-specific CD8**^+^** T cells gain memory cell properties (40). During the development of tumor-reactive CD8**^+^** T cells against sporadic cancer, a premalignant phase has been described that has the potential of priming T cell reactivity in a noninflammatory context (14, 16). Hence, lack of this inflammatory environment putatively could lead to a deviation from CD8^+^ T cell differentiation processes during infection. Experimentally, we largely excluded an inflammatory stimulus through our immunization procedure.

To explore a link among precursor frequency, avidity, and differentiation potential, we made use of the MataHari TCR-transgenic strain. In contrast to the high-avidity OT-I CD8**^+^** T cells recognizing a strong neoantigen from ovalbumin, adoptive MataHari T cell transfers required more naive precursors to visualize a distinct CD8**^+^** memory population (41). In keeping with the polyclonal HY-reactive repertoire, CD8**^+^** T cell expansion and differentiation processes in the effector phase were indistinguishable between *Ebag9*^+/+^ and *Ebag9*^–/–^ MataHari cells. However, in the memory phase, transferred *Ebag9*^–/–^ MataHari cells expanded to higher numbers and frequencies and, therefore, recapitulated the polyclonal response. We conclude that a monoclonal TCR population, as compared with a polyclonal naive repertoire, favored a similarly biased memory differentiation when *Ebag9* was deleted. Thus, TCR avidity, although playing a role in clonal selection during the response primed under inflammatory conditions (42), did not alter fate decisions when the cytolytic capacity was high and inflammation was reduced. Our results are in agreement with gene expression studies showing that the frequencies of antigen-specific T cell precursors and TCR repertoire did not greatly alter gene expression at effector or memory time points (43).

When mice were challenged with the strongly immunogenic TAg antigen, we could essentially recapitulate activation and memory differentiation processes as seen in the HY-antigen model. In the memory phase, *Ebag9*^–/–^ mice developed an expanded TAg-specific CD8^+^ T cell pool, suggesting that Ebag9-mediated effects on CD8^+^ T cell fate decisions were not limited to a particular miHAg model in which the TCR signal strength was probably different from those in the TAg-reactive population.

In context of infection models, the strength and duration of infection are implicated in acquisition of a memory fate. Curtailing the duration of antigen availability promotes the differentiation of memory (44). In our immunization system, which lacked a strong inflammatory stimulus and exposed animals to identical antigenic challenges, the only variable was, instead, the cytolytic capacity of CTLs (11, 12). We suggest that the extent of cytolysis by a common pool of CTLs regulated the preferred differentiation into memory cells.

scRNA-Seq analysis of TAg-antigen specific CD8^+^ T cells supported our interpretation of the regulation of memory differentiation. Although we did not reveal profound transcriptional differences between WT and *Ebag9*^–/–^ CTLs regarding transcriptional, epigenetic, or metabolic regulators, we determined that differences in memory responses were reflected by changes in the frequencies of cells in subpopulations. Strikingly, an increased cluster 1 in *Ebag9*^–/–^ CTLs characterized by a modest overexpression of *Id3*, but not its counter-regulatory partner, *Id2*, would be consistent with a precursor memory signature. For the HY-antigen model, at day 10, essentially the same imbalance between *Id2* and *Id3* was obtained in Ebag9^–/–^ CTLs, supporting our notion in the TAg-antigen model.

Id3^hi^ precursors of long-lived memory cells could be identified before the peak of T cell population expansion or upregulation of cell-surface receptors that indicate memory potential (45, 46). Although the *Id3* increase seemed rather small, even in infection models, at the peak of the primary response, T-bet expression between terminal effector and precursor memory cells differed only 2-fold (22), indicating that the differences observed here are sufficient for preferential fate decisions.

In *Ebag9*^–/–^ subsets, clusters 0, 1, and 8 had higher expression of memory-related transcription factors *Zeb1*, *Id3*, and *Stat3*. The transcription factor *Zeb1* represses *Il2* and is critical for memory CD8^+^ T cell survival and function (47). Activation of the signal transducer and activator of transcription 3 (*Stat3*) promotes the expression of Bcl-6 that enhances memory CD8^+^ T cell differentiation (48). Interestingly, the memory cluster 0 and the precursor memory cluster 1 in WT CD8^+^ T cells had higher gene expression of the pro-apoptotic factor *Bbc3*. In contrast, there was greater expression of anti-apoptotic genes associated with the NF-κB pathway in clusters 8 and 1 of *Ebag9^–/–^* T cells (34, 35). The greater gene expression in those T cell clusters might enable those cells to escape activation-induced cell death mediated by cytolytic effector molecules and to form a memory pool.

Differentiation models for the in vivo generation of memory T cell subsets differ mainly with respect to the proposed timing of memory T cell development during the expansion phase (49). According to the signal-strength model and, alternatively, the decreasing-potential model (3), repetitive stimulation of T cells with antigen and proinflammatory cytokines induces greater proliferation and terminal effector cell differentiation. However, truncating the duration of antigen exposure and limiting the inflammatory co-signal instead promotes memory precursor T cell formation. From our model, we infer that *Ebag9^–/–^* CD8^+^ T cells have a greater cytolytic capacity, which leads to a faster antigen removal and, thus, truncation of the antigenic stimulus. Hence, weak signals on a population level would allow more latecomers to acquire memory differentiation and, conversely, fewer signals progressively driving terminal effector differentiation. We suggest that EBAG9 defines a crucial regulator for the cytolytic capacity of CD8^+^ T cells and their memory fate decisions. Hence, deleting *Ebag*9 in engineered T cells might be suitable for therapeutic exploitation, foremost in adoptive T cell transfer targeted at hematopoietic neoplasm.

In summary, the *Ebag*9-associated cytolytic enhancer model provided insight into CD8^+^ T cell programming, either when stimulated briefly or when challenged by a chronic leukemia burden.

## Methods

### Mice.

*Ebag9*^–/–^ mice were generated as described (11) and backcrossed into B6 mice for 14 generations. C57BL/6 (CD45.2) mice were bred in-house; CD45.1 congenic B6 mice were obtained from Charles River Laboratories; and CD90.1 (Thy1.1; B6) congenic mice were obtained from Taconic Biosciences. MataHari TCR transgenic mice (C57BL/6 background) were provided by Il-Kang Na (Charité – Universitätsmedizin Berlin, Berlin, Germany; ref. 28); these mice exhibit an HY-specific TCR that recognizes the D^b^-restricted peptide WMHHNMLDI derived from the *Uty* gene. The *Ebag9*^–/–^ strain was backcrossed into transgenic MataHari mice to obtain *Ebag9*^–/–^ MataHari double-transgenic animals for another 4–5 generations. Heterozygous littermates were bred to obtain *Ebag9*^+/+^ × MataHari mice. These strains are also referred to as MataHari^+/+^ or MataHari^–/–^, respectively.

*Ebag9*^–/–^ mice were backcrossed into transgenic *Eμ-Tcl1* mice (17) (at least 10 backcrosses onto a C57BL/6 background) to obtain *Eμ-Tcl1 Ebag9*^–/–^, *Eμ-Tcl1 Ebag9*^+/+^**, and *Eμ-Tcl1 Ebag9*^+/–^ double-transgenic animals. This strain develops B cell CLL that can be detected by flow cytometry analysis in blood and spleen at the earliest at 2 months of age (18). The strain was provided by Carlo Croce (The Ohio State University Cancer Center [OSUCC], Columbus, Ohio, USA). All mice were maintained and bred under standard pathogen-free conditions at the animal core facility of the Max Delbrück Center for Molecular Medicine. Light cycles were at 12-hour intervals, temperature was kept at 22°C, and humidity was kept at 55%, in compliance with the institutional rules. Female mice between the ages of 8 and 10 weeks were used as recipients for adoptive transfer or immunization experiments.

### Generation of mixed BM chimeras.

CD45.1 (Thy1.2) female recipient mice were lethally irradiated with 9–9.5 Gy split in 2 doses. BM cells from female congenic donors (CD45.2, Thy1.1, or Thy1.2) were obtained by flushing femurs and tibias with RPMI medium. Cells from 2–3 donors for each genotype were pooled and adjusted to 2.5 × 10^6^ cells/50 μL in RPMI medium. Congenic WT- and *Ebag9*^–/–^-derived BM cells were then mixed in a 1:1 ratio and injected i.v. at 5 × 10^6^/100 μL cells into congenic (CD45.1 Thy1.2) recipient mice. At weeks 6 and 8, mice were immunized i.p. with 5 × 10^6^ male B6 splenocytes. Animals were sacrificed 6–7 weeks after the last immunization, and splenocytes were analyzed by flow cytometry for the presence of HY-reactive congenic-donor CD8^+^ T cells.

### Adoptive T cell transfer.

For transfer of monoclonal CD8^+^ T cells specific for the HY-antigen, splenocytes from female MataHari^+/+^ and MataHari^–/–^ (both CD45.2^+^CD90.2^+^) mice were enriched with a MACS bead CD8^+^ T cell negative selection kit (Miltenyi Biotec). Cells from 3 animals per genotype were pooled for each assay. To obtain naive transgenic CD8^+^ T cells, the enriched fraction was further stained for CD8^+^, TCR Vβ 8.3^+^, and CD62L^hi^ expression, followed by flow cytometry sorting on a FACSAria 3 or FACSAria Fusion instrument (Becton Dickinson). Sorted CD8^+^ T cells were transferred i.v. at 4 × 10^4^ to 5 × 10^4^ cells into congenic recipient female mice (CD45.1). Where indicated, CD90.1 congenic recipient mice were used. One day after transfer, mice were immunized with 5 × 10^6^ B6 male splenocytes i.p. in RPMI medium. Transferred congenic CD8^+^CD45.2^+^ and congenic CD8^+^CD90.2^+^ T cells were analyzed in the effector phase at day 7, and memory formation was studied more than 50 days after immunization.

### Flow cytometry.

Single-cell suspensions of splenocytes were blocked with anti–CD16/CD32 Abs in flow cytometry buffer (PBS, 0.5% BSA, 0.05% NaN_3_), followed by staining with the indicated Abs. Appropriate isotype controls were always included. For the detection of HY-antigen–specific CD8^+^ T cells, a phycoerythrin-coupled (PE-coupled) D^b^/WMHHNMLDI Pentamer (ProImmune) or an APC-conjugated D^b^/WMHHNMLDI Dextramer (Immudex) were used and are referred to in this article as HY-Pentamer or HY-Dextramer. To identify TAg-specific CD8^+^ T cells, PE-labeled K^b^/pIV dextramers or tetramers (MBL International Corporation or Immudex) were used. In context of the K^b^-restriction element, peptide IV (VVYDFLKC) is the immunodominant epitope among the SV-40 large TAg-derived antigens. Where indicated, a negative selection MACS bead kit (Miltenyi Biotec) was used for the enrichment of CD8^+^ T cells prior to Ab or multimer staining. In some experiments for the assessment of the anti-HY CD8^+^ memory response, CD8^+^ splenocytes were co-cultivated for 60 hours with peptide-pulsed (WMHHNMLDI), BM-derived DCs. DCs were generated as described (50).

Abs and fixation kits used for flow cytometry are listed in Supplemental Table 3. Analytical samples were acquired on a FACSCanto II flow cytometer (BD Biosciences) and analyzed with FlowJo software.

### Data availability.

scRNA-Seq data have been deposited in NCBI’s Gene Expression Omnibus (GEO) (51) and are accessible through GEO Series accession number GSE156611 (https://www.ncbi.nlm.nih.gov/geo/query/acc.cgi?acc= GSE156611). All data are available in the main text or the supplemental materials.

### Statistics.

Results are expressed as the arithmetic means ± SEM. *P* < 0.05 was considered statistically significant, as determined by the 2-tailed Mann-Whitney test for non-normally distributed data, or the 2-tailed paired and unpaired Student’s *t* test for normally distributed data and large sample sizes, where appropriate.

### Study approval.

Animal studies were performed according to institutional and Berlin State guidelines (approved by the Landesamt für Gesundheit und Soziales, Berlin; approval TVV G 0089/10, G 0091/15, and G 0373/13).

More detailed methods are provided in Supplemental Methods.

## Author contributions

AR conceived and served as project administrator for the study. AW, KG, SH, DH, CF, SS, and GW contributed to the study methodology. AW, KG, SH, DH, CF, AR, and UEH contributed to the investigations. Visualization was performed by UEH, AW, and AR. AR and UEH acquired funding for the study and supervised the study. AR and AW wrote the original draft of the manuscript; UEH, AR, AW, SH, KG, CF, SS, and GW reviewed and edited the manuscript. AR and AW contributed equally as co–first authors to this study. Both performed key experiments, wrote the original draft of the paper, analyzed the data, and established the methodology. AR, who conceived and planned the study, is listed first.

## Supplementary Material

Supplemental data

## Version 1 04/28/2022

In-Press Preview

## Version 2 06/08/2022

Electronic publication

## References

[1] Russell JH, Ley TJ (2002). Lymphocyte-mediated cytotoxicity. Annu Rev Immunol.

[2] Voskoboinik I (2015). Perforin and granzymes: function, dysfunction and human pathology. Nat Rev Immunol.

[3] Kaech SM, Cui W (2012). Transcriptional control of effector and memory CD8+ T cell differentiation. Nat Rev Immunol.

[4] Kaech SM (2003). Selective expression of the interleukin 7 receptor identifies effector CD8 T cells that give rise to long-lived memory cells. Nat Immunol.

[5] Youngblood B (2013). T-cell memory differentiation: insights from transcriptional signatures and epigenetics. Immunology.

[6] Khan SH, Badovinac VP (2015). Listeria monocytogenes: a model pathogen to study antigen-specific memory CD8 T cell responses. Semin Immunopathol.

[7] Lefrancois L, Obar JJ (2010). Once a killer, always a killer: from cytotoxic T cell to memory cell. Immunol Rev.

[8] Harty JT, Badovinac VP (2008). Shaping and reshaping CD8+ T-cell memory. Nat Rev Immunol.

[9] Pipkin ME (2010). Interleukin-2 and inflammation induce distinct transcriptional programs that promote the differentiation of effector cytolytic T cells. Immunity.

[10] Schluns KS, Lefrancois L (2003). Cytokine control of memory T-cell development and survival. Nat Rev Immunol.

[11] Ruder C (2009). The tumor-associated antigen EBAG9 negatively regulates the cytolytic capacity of mouse CD8+ T cells. J Clin Invest.

[12] Miyazaki T (2014). EBAG9 modulates host immune defense against tumor formation and metastasis by regulating cytotoxic activity of T lymphocytes. Oncogenesis.

[13] Harty JT, Badovinac VP (2002). Influence of effector molecules on the CD8(+) T cell response to infection. Curr Opin Immunol.

[14] Schietinger A (2016). Tumor-specific T cell dysfunction is a dynamic antigen-driven differentiation program initiated early during tumorigenesis. Immunity.

[15] McLane LM (2019). CD8 T cell exhaustion during chronic viral infection and cancer. Annu Rev Immunol.

[16] Willimsky G (2008). Immunogenicity of premalignant lesions is the primary cause of general cytotoxic T lymphocyte unresponsiveness. J Exp Med.

[17] Bichi R (2002). Human chronic lymphocytic leukemia modeled in mouse by targeted TCL1 expression. Proc Natl Acad Sci U S A.

[18] Heinig K (2014). Access to follicular dendritic cells is a pivotal step in murine chronic lymphocytic leukemia B-cell activation and proliferation. Cancer Discov.

[19] Ramsay AG (2008). Chronic lymphocytic leukemia T cells show impaired immunological synapse formation that can be reversed with an immunomodulating drug. J Clin Invest.

[20] Riches JC (2013). T cells from CLL patients exhibit features of T-cell exhaustion but retain capacity for cytokine production. Blood.

[21] Riddell SR (2002). T-cell therapy of leukemia. Cancer Control.

[22] Joshi NS (2007). Inflammation directs memory precursor and short-lived effector CD8(+) T cell fates via the graded expression of T-bet transcription factor. Immunity.

[23] Millrain M (2001). Examination of HY response: T cell expansion, immunodominance, and cross-priming revealed by HY tetramer analysis. J Immunol.

[24] Hendriks J (2000). CD27 is required for generation and long-term maintenance of T cell immunity. Nat Immunol.

[25] Marzo AL (2005). Initial T cell frequency dictates memory CD8+ T cell lineage commitment. Nat Immunol.

[26] Tada H (1986). An improved colorimetric assay for interleukin 2. J Immunol Methods.

[27] Karim M (2005). CD25+CD4+ regulatory T cells generated by exposure to a model protein antigen prevent allograft rejection: antigen-specific reactivation in vivo is critical for bystander regulation. Blood.

[28] Valujskikh A (2002). Cross-primed CD8(+) T cells mediate graft rejection via a distinct effector pathway. Nat Immunol.

[29] Willimsky G, Blankenstein T (2007). The adaptive immune response to sporadic cancer. Immunol Rev.

[30] Butler A (2018). Integrating single-cell transcriptomic data across different conditions, technologies, and species. Nat Biotechnol.

[31] Liberzon A (2015). The molecular signatures database (MSigDB) hallmark gene set collection. Cell Syst.

[32] Bergen V (2020). Generalizing RNA velocity to transient cell states through dynamical modeling. Nat Biotechnol.

[33] Speiser DE (1997). A regulatory role for TRAF1 in antigen-induced apoptosis of T cells. J Exp Med.

[34] Barkett M, Gilmore TD (1999). Control of apoptosis by Rel/NF-kappaB transcription factors. Oncogene.

[35] Mondor I (2005). RelA regulates the survival of activated effector CD8 T cells. Cell Death Differ.

[36] Man K, Kallies A (2015). Synchronizing transcriptional control of T cell metabolism and function. Nat Rev Immunol.

[37] Kaech SM (2002). Effector and memory T-cell differentiation: implications for vaccine development. Nat Rev Immunol.

[38] McClanahan F (2015). PD-L1 checkpoint blockade prevents immune dysfunction and leukemia development in a mouse model of chronic lymphocytic leukemia. Blood.

[39] McClanahan F (2015). Mechanisms of PD-L1/PD-1-mediated CD8 T-cell dysfunction in the context of aging-related immune defects in the E*μ*-TCL1 CLL mouse model. Blood.

[40] Starbeck-Miller GR (2014). IL-12 and type I interferon prolong the division of activated CD8 T cells by maintaining high-affinity IL-2 signaling in vivo. J Exp Med.

[41] Turner MJ (2008). Avidity maturation of memory CD8 T cells is limited by self-antigen expression. J Exp Med.

[42] Plumlee CR (2013). Environmental cues dictate the fate of individual CD8+ T cells responding to infection. Immunity.

[43] Best JA (2013). Transcriptional insights into the CD8(+) T cell response to infection and memory T cell formation. Nat Immunol.

[44] Zehn D (2009). Complete but curtailed T-cell response to very low-affinity antigen. Nature.

[45] Yang CY (2011). The transcriptional regulators Id2 and Id3 control the formation of distinct memory CD8+ T cell subsets. Nat Immunol.

[46] Ji Y (2011). Repression of the DNA-binding inhibitor Id3 by Blimp-1 limits the formation of memory CD8+ T cells. Nat Immunol.

[47] Guan T (2018). ZEB1, ZEB2, and the miR-200 family form a counterregulatory network to regulate CD8^+^ T cell fates. J Exp Med.

[48] Cui W (2011). An interleukin-21-interleukin-10-STAT3 pathway is critical for functional maturation of memory CD8+ T cells. Immunity.

[49] Ahmed R (2009). The precursors of memory: models and controversies. Nat Rev Immunol.

[50] Rehm A (2014). Dendritic cell-mediated survival signals in E*μ*-Myc B-cell lymphoma depend on the transcription factor C/EBPβ. Nat Commun.

[51] Edgar R (2002). Gene expression omnibus: NCBI gene expression and hybridization array data repository. Nucleic Acids Res.

