# Supplementary material for: EBAG9 controls CD8+ T cell memory formation responding to tumor challenge in mice
Source: JCI Insight. 2022 Jun 8;7(11):e155534. doi: 10.1172/jci.insight.155534 (PMC9220939; doi:10.1172/jci.insight.155534)
Supplement: Supplemental data [file jciinsight-7-155534-s045.pdf]

Inventory of Supplementary material

Supplementary Figures S1-S7

Supplementary Table S1, S2

Supplementary Figure legends

Supplementary Material and Methods

Supplementary References

Supplementary Figure S1

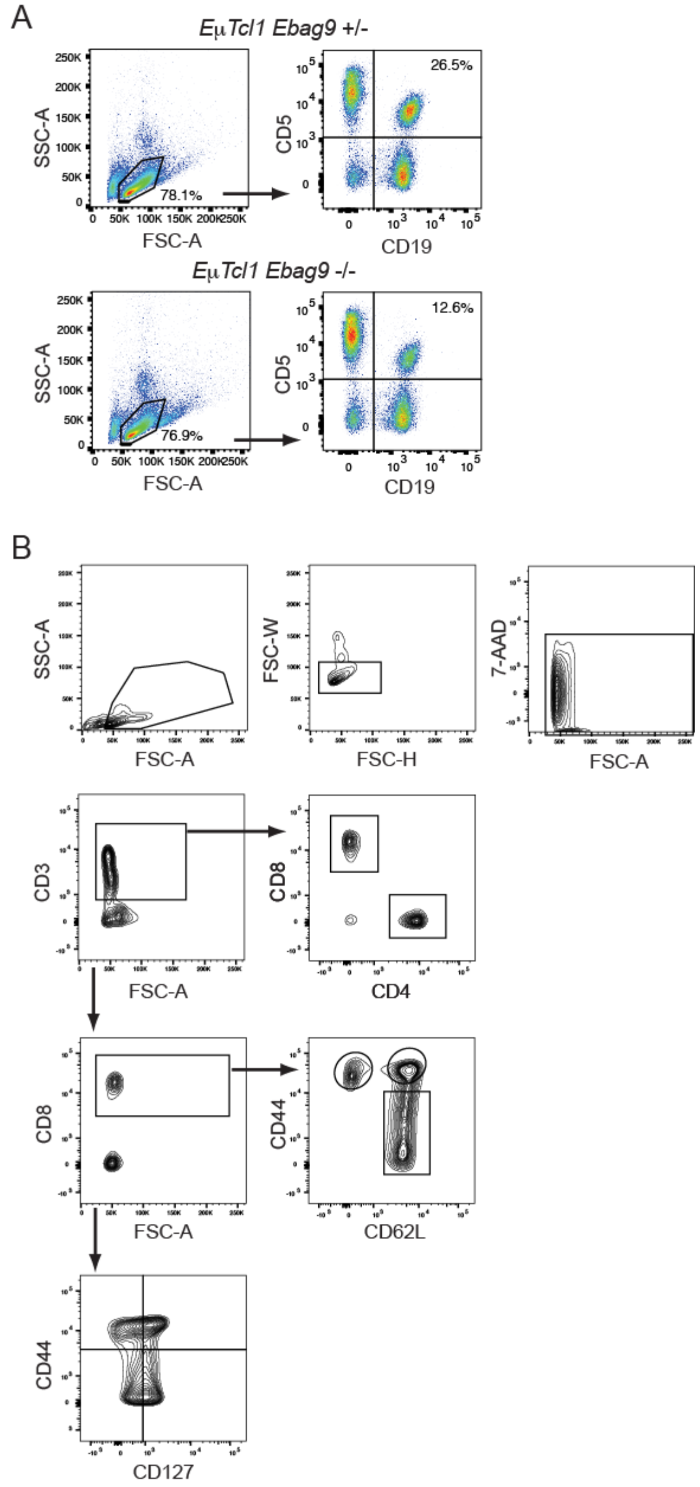

Figure S1. Representative FACS plots for leukemia cell gating and T cell subset classification. (**A**) Flow cytometry gating strategy to characterize and quantitate leukemia *Eμ-Tcl1* cell frequencies. Leukemic cells derived from spleen and PB were

characterized as $CD19^+CD5^{int}$ cells in contrast to $CD19^-CD5^+$ T cells (T), $CD19^+CD5^-$ follicular B cells. Numbers on the gates (upper right) are the percentages of $CD19^+CD5^{int}$ cells, calculated based on all cells within the lymphocyte gate. Exemplary, splenic cells derived from diseased *Ebag9*$^{+/-}$ and *Ebag9*$^{-/-}$ x*Eμ-Tcl1* transgenic mice are depicted here. **(B**) Representative gating strategy for T cell subsets within spleens of diseased mice. Gates were set first for lymphocytes, exclusion of doublets, and viability 7-AAD$^-$. T cells were identified by $CD3^+CD4^+$ and $CD3^+CD8^+$ staining. Activation and memory status refers to $CD3^+CD8^+$ T cells only. Further markers used were CD44, CD62L, and CD127 to characterize antigen-experienced, naive and memory subsets.

Supplementary Figure S2

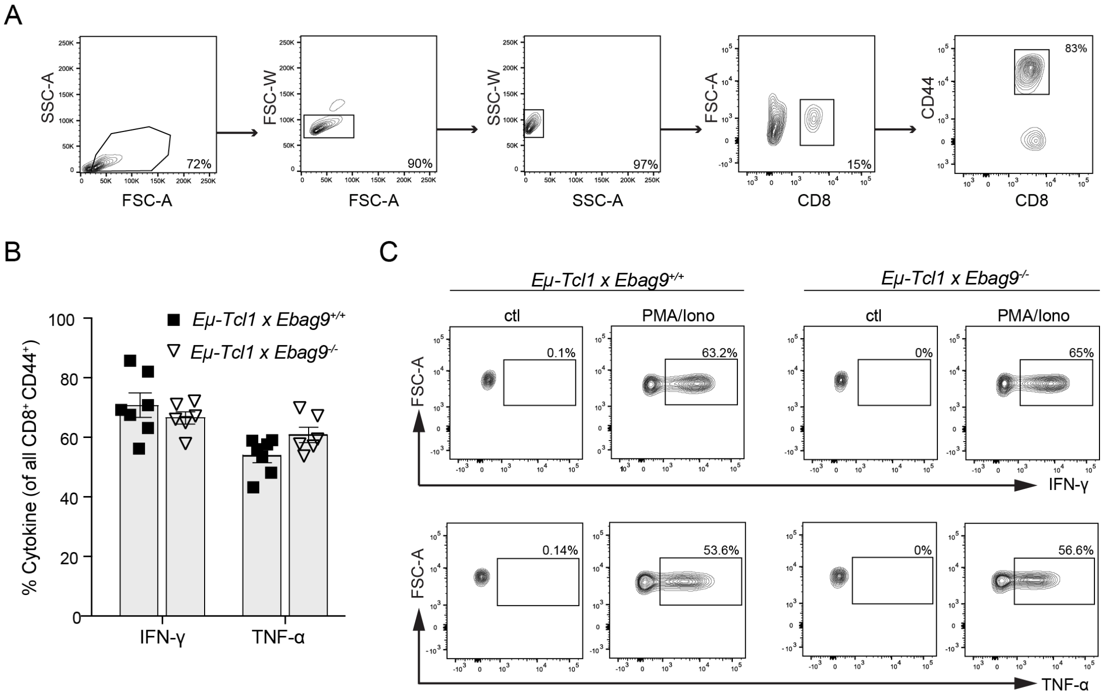

Figure S2. Ebag9-deficiency does not affect cytokine production in tumor-experienced CD8$^+$ T cells. Cryopreserved CLL-bearing splenocytes isolated from *Eμ-Tcl1xEbag9*$^{+/-}$ and *Eμ-Tcl1xEbag9*$^{-/-}$ mice were thawed and stimulated for 4 h with PMA and Ionomycin in the presence of Brefeldin-A. Activated CD44$^+$ CD8$^+$ T cells were analyzed for IFN-γ and TNF-α expression by intracellular staining. **(A)** All steps of a representative gating strategy for FACS analysis are given. **(B)** Bar graph shows percentages ofcytokine expressing activated CD8$^+$ CD44$^+$ T cells. Bars indicate mean values ± SEM; n=3 independent experiments with n=6 animals per group. **(C)** Representative dot plots are shown. Numbers in the gates are the percentages of cytokine-containing activated CD8$^+$ T cells.

Supplementary Figure S3

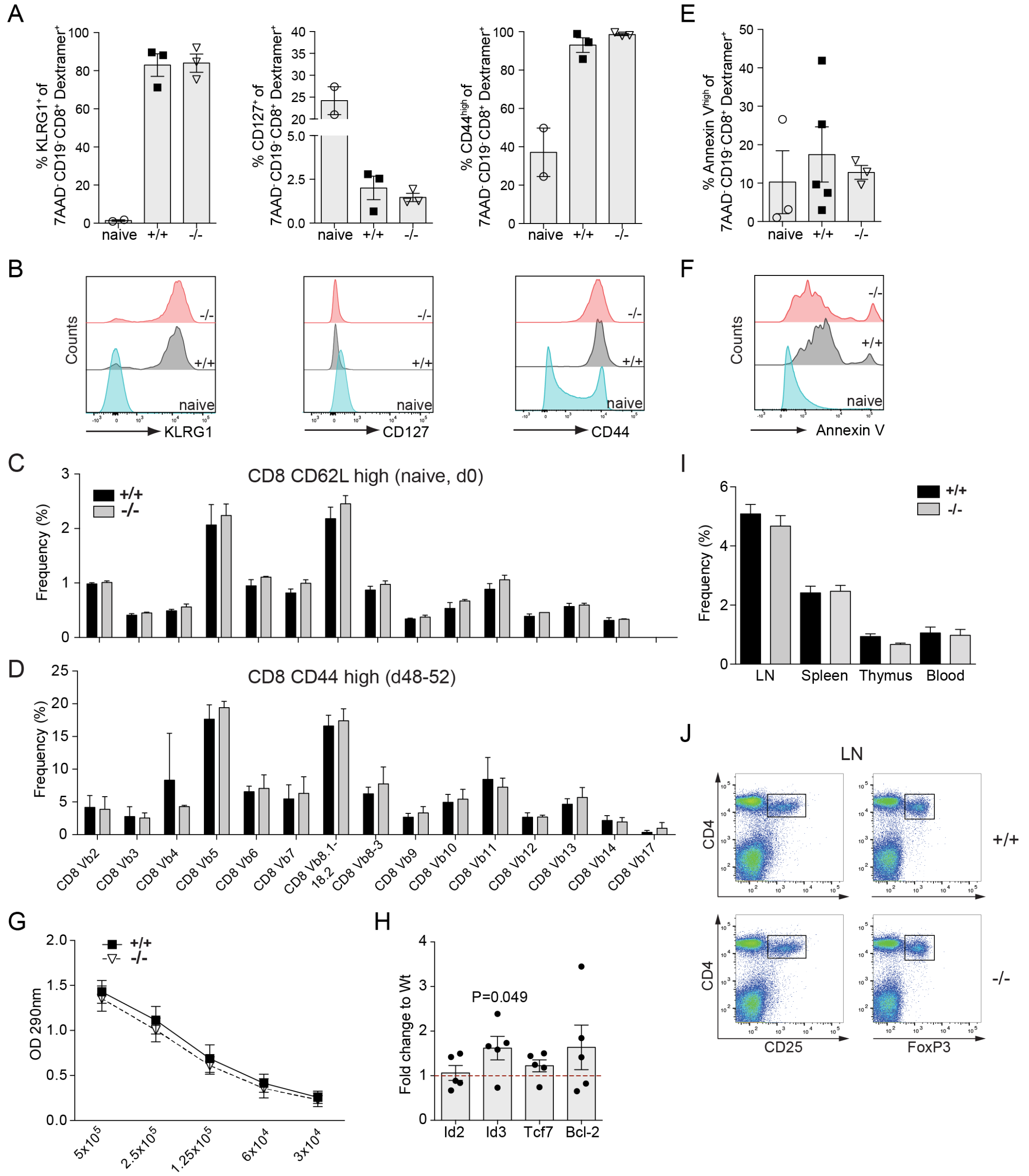

Figure S3. Activation and differentiation processes in the effector phase of an anti-HY response in Wt and Ebag9$^{-/-}$ mice cannot be distinguished by phenotypic markers. (A) Female Wt (B6) and Ebag9$^{-/-}$ mice were immunized twice i.p. with 5x10^{6} male splenocytes (B6), as shown as shown in Figure 2A. At day 11, splenocytes were isolated, and T lymphocytes were stained with anti-CD8 and Dextramer/HY, followed by flow cytometry analysis. Activation and differentiation

of this gated population (Figure 2C) was further assessed by anti-CD44 staining; terminal effector cells exhibit KLRG1 reactivity, and the precursor memory population was defined by anti-CD127 staining. Bars show mean values ± SEM. Data are representative of n=2 experiments, with n=2 (naïve) and n=3 (Wt and *Ebag9*$^{-/-}$) mice per group. **(B)** Representative overlay histograms show shifts relative to CD8^{+} naïve T cells. (**C**) Ebag9-deficiency does not affect the TCR repertoire in naive CD8^{+} T cells. Total splenocytes from naive Wt (n=6) and Ebag9$^{-/-}$ mice (n=6) were stained with CD8, CD62L and TCR Vβ-specific antibodies; n=3 independent experiments. The bar graph indicates the mean ± SEM frequencies in percent of Vβ chain-expressing naive CD8^{+} CD62L^{high} T lymphocytes. (**D**) The TCR repertoire in antigen-experienced Wt and Ebag9$^{-/-}$ CD8^{+} T cells is indistinguishable. Female Wt (n=4) and Ebag9$^{-/-}$ mice (n=4) were immunized twice i.p. with 5x10^{6} male splenocytes. At day 48-52, splenic CD8^{+} T cells were MACS isolated and re-stimulated *in vitro* for 3 days with 1 μg/ml HY-peptide-pulsed DCs. Activated CD8^{+} T cells were detected with anti-CD44 staining, and the TCR repertoire was further differentiated with anti-TCR Vβ antibodies. Bar graphs indicate the mean values ± SEM of the Vβ chain frequencies in percent in antigen-experienced CD8^{+} CD44^{high} T lymphocytes. n= 2 independent experiments. In (**C**) and (**D**), a Mann-Whitney test was applied.

(E) Mice were immunized as in **(A),** and T lymphocytes were stained with anti**-**CD8, Dextramer/HY and annexin V, followed by flow cytometry analysis. Bars show mean values ± SEM. Data are representative of n=2 experiments, with n=2 (naïve) and n=3 (Wt and *Ebag9*$^{-/-}$) mice per group. **(F)** Representative overlay histogram show shifts relative to CD8^{+} naïve T cells.

(**G**) Anti-HY reactive $CD8^+$ T cells proliferate at the same rate. Female Wt and *Ebag9*$^{-/-}$ splenocytes were stimulated with irradiated male Wt splenocytes in a mixed lymphocyte reaction over 5 days, followed by a CD8 MACS bead negative selection. $CD8^+$ lymphocytes were then titrated and cultured for 4 hrs. Conversion of a tetrazolium salt into a formazan product in cell culture was recorded as the absorbance at 490 nm. Assay was performed 2 times, with n=6 animals per genotype. Graph indicates mean values ±SEM for each dilution step. **(H)** Female Wt and Ebag9$^{-/-}$ mice were immunized as in **(A)**. At day 11, splenic $CD8^+$ Pentamer/HY^+ T cells were sorted by flow cytometry and analyzed for relative mRNA levels of the indicated genes by RT-qPCR. Gene expression levels were normalized to Wt Pentamer/HY-reactive $CD8^+$ T cells, set arbitrarily to 1 (dashed red line). Fold changes in mRNA are shown as mean values ± SEM. Data are representative of n=5-6 independent experiments with RNA from $CD8^+$ T cells, pooled from 2-3 mice per condition. A Student's t-test was applied; *p<0,05.

(**I**) Under homeostatic conditions, Wt and Ebag9$^{-/-}$ mice have similar frequencies of regulatory T cells. Percentages of $Foxp3^+$ cells from gated $CD4^+$ $CD25^+$ lymphocytes in lymph nodes (LN), spleen, thymus and peripheral blood; n=5 independent experiments with Wt, n=14 and *Ebag9*$^{-/-}$, n=16 animals. Data represent the mean values ± SEM. (**J**) $Foxp3^+$ regulatory T cells are not altered in Ebag9$^{-/-}$ naive mice. Representative dot plots for the flow cytometry analysis of $CD4^+CD25^+$ and $CD4^+$ $FoxP3^+$ regulatory T cells in spleen and lymph nodes (LN). Cells were stained and analyzed by flow cytometry directly after isolation.

Supplementary Figure S4

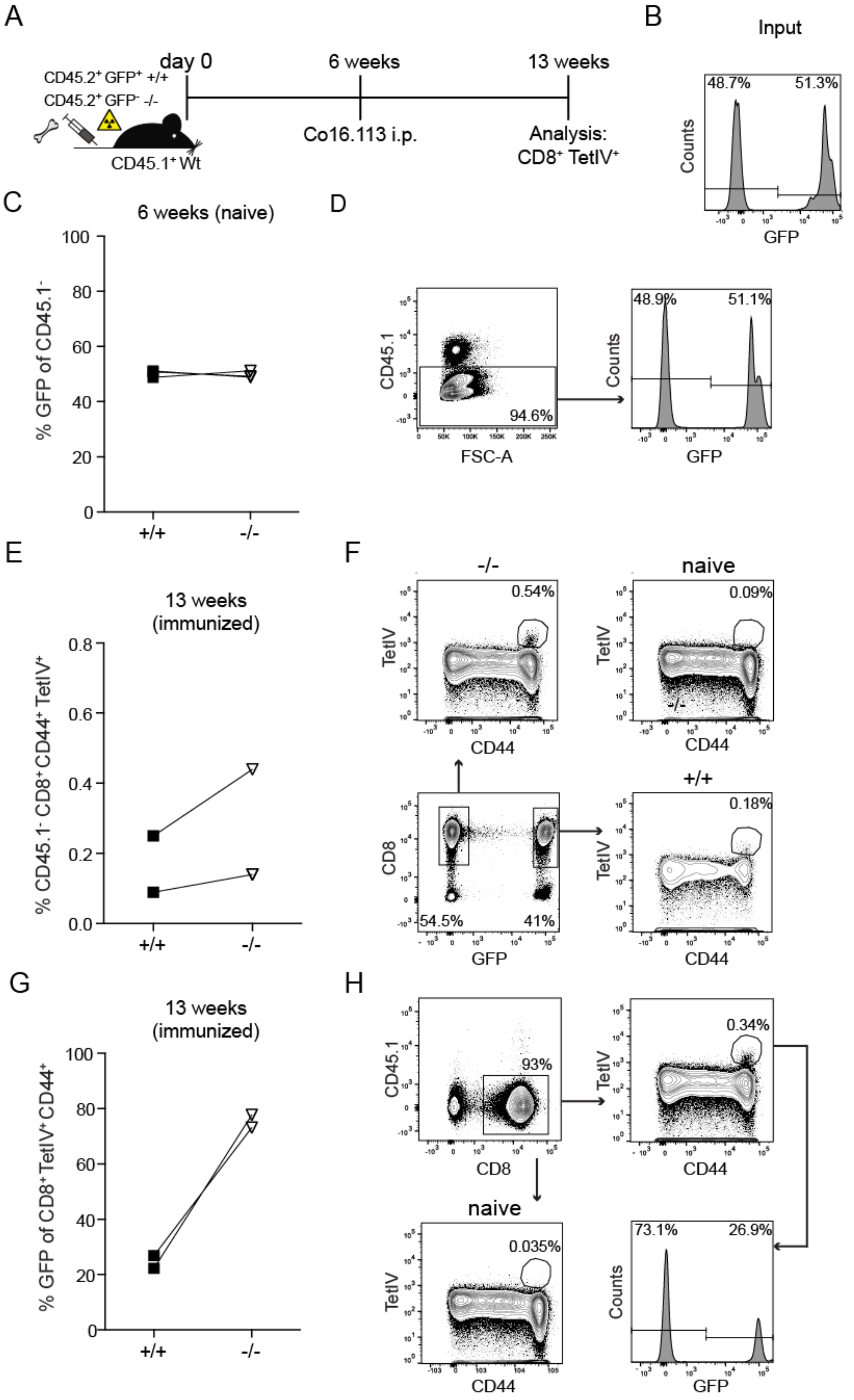

Figure S4. The predominance of *Ebag9*$^{-/-}$-derived antigen-specific memory CD8$^+$ T cells is not driven by a preferential engraftment of *Ebag9*$^{-/-}$ bone marrow. (A) CD45.1$^+$ congenic recipient mice were lethally irradiated and transplanted with a 1:1 mixture of 2.5x10^6 CD45.2$^+$ Ubc-GFP$^+$ (Wt, Ebag9$^{+/+}$) and 2.5x10^6 CD45.2$^+$ Ubc-GFP$^-$ (Ebag9$^{-/-}$) BM cells. After 6 weeks, chimeric mice were either analyzed (=naïve) directly, or immunized i.p. with 1.5x10^6 Tag-expressing Co16.113 tumor cells. Seven weeks later, splenic CD8$^+$ T cells were MACS-bead enriched and analyzed without further re-stimulation; Wt, GFP$^+$; Ebag9$^{-/-}$, GFP$^-$. **(B)** Histogram of 1:1 mixed bone marrow before transfer into recipient mice is shown. **(C)** Equal engraftment of Wt-derived GFP$^+$ and *Ebag9*$^{-/-}$-derived GFP$^-$ bone marrow is shown. Mice were analyzed 6 weeks after bone marrow transfer: Residual CD45.1$^+$ host cells were rigorously excluded. Data of n=3 mice are shown. **(D)** The gating strategy for **(C)** is given. **(E)** Differentiation of congenic donor Wt (+/+) and *Ebag9*$^{-/-}$ (-/-) CD8$^+$ T cells by GFP expression. Residual CD45.1$^+$ host cells were excluded. Analysis of TAg-specific T cells by TetIV-staining. Graph depicts the frequencies of CD44$^+$ TetIV$^+$ CD8$^+$ T cells among the GFP$^+$ (Wt) and GFP$^-$ (*Ebag9*$^{-/-}$) CD8$^+$ T cell population in single recipient mice. Data of n=2 mice are shown. **(F)** The gating strategy for detection of CD8$^+$ CD44$^+$ TetIV$^+$ T cells within the gates GFP$^+$ and GFP$^-$ population is shown. **(G)** Predominance of *Ebag9*$^{-/-}$-deficient Tag-reactive T cells over Wt cells is shown. Depicted are the percentages of TetIV$^+$ CD44$^+$ CD8$^+$ T cells derived from Wt (GFP$^+$) or *Ebag9*$^{-/-}$ (GFP$^-$) progenies from n=2 mice. **(H)** Representative FACS dot plots for the detection of CD45.1$^-$ CD8$^+$ CD44$^+$ TetIV$^+$ T cells are shown.

Supplementary Figure S5

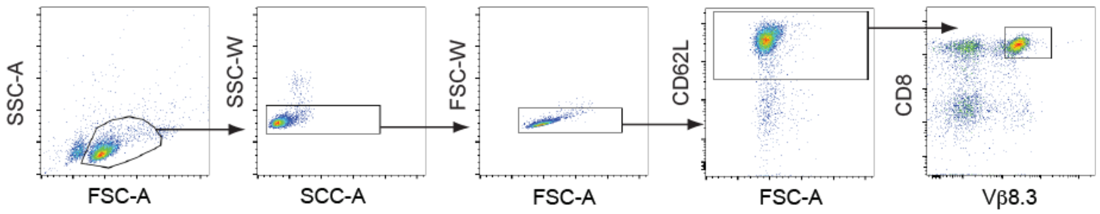

Figure S5. A representative dot plot for the gating of MataHari CD8$^+$ transgenic T cells. Splenocytes from Ebag9$^{+/+}$ x MataHari and Ebag9$^{-/-}$ x MataHari mice were first enriched by MACS CD8 bead sorting, followed by staining for CD8, TCR Vβ8.3 and CD62L. All steps of a representative gating strategy for FACS sorting are given. Transplanted cells were CD8$^+$CD62L^{high}Vβ8.3$^+$.

Supplementary Figure S6

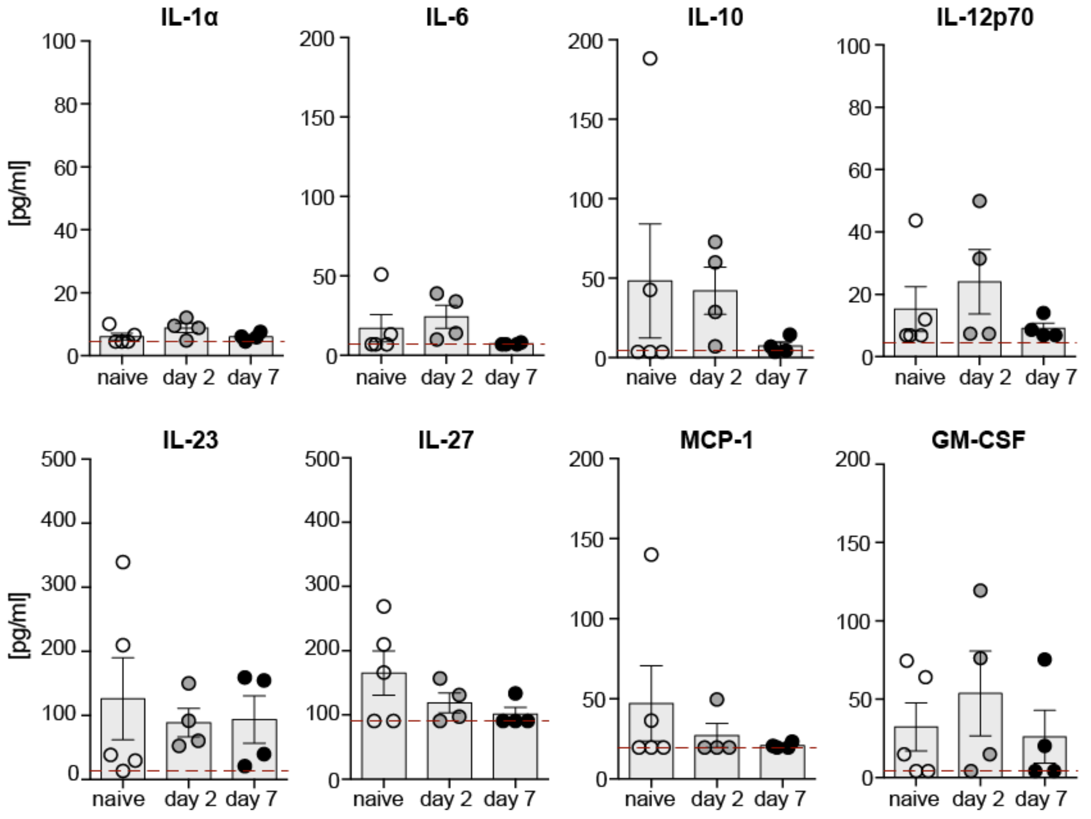

Figure S6. Non-inflammatory conditions prevail after immunization with a low dose of TAg-expressing tumor cell line Co16.113. Mice were immunized i.p. with $1.5x10^6$ Co16.113 tumor cells or with sterile RPMI media. At day 2 and 7 after immunization, serum samples from heart punctures were collected and analyzed by FACS-basedbead array for inflammatory cytokines. Bars represent sample concentrations in [pg/ml] at the indicated condition and time point for n=5 (naïve) and n=4 (day 2 and 7) mice. The dashed red line represents the detection threshold of the bead array (IL-1α: 4.55 pg/ml; IL-6: 6.58 pg/ml; IL-10: 3.52 pg/ml; IL-12p70: 6.88 pg/ml; IL-23: 13.72 pg/ml; IL-27: 90.65 pg/ml; MCP-1: 19.53 pg/ml; GM-CSF: 4.28 pg/ml). For IFN-β, IFN-γ, TNF-α, IL-1β and IL-17a all sample concentrations were below the detection threshold (data not shown).

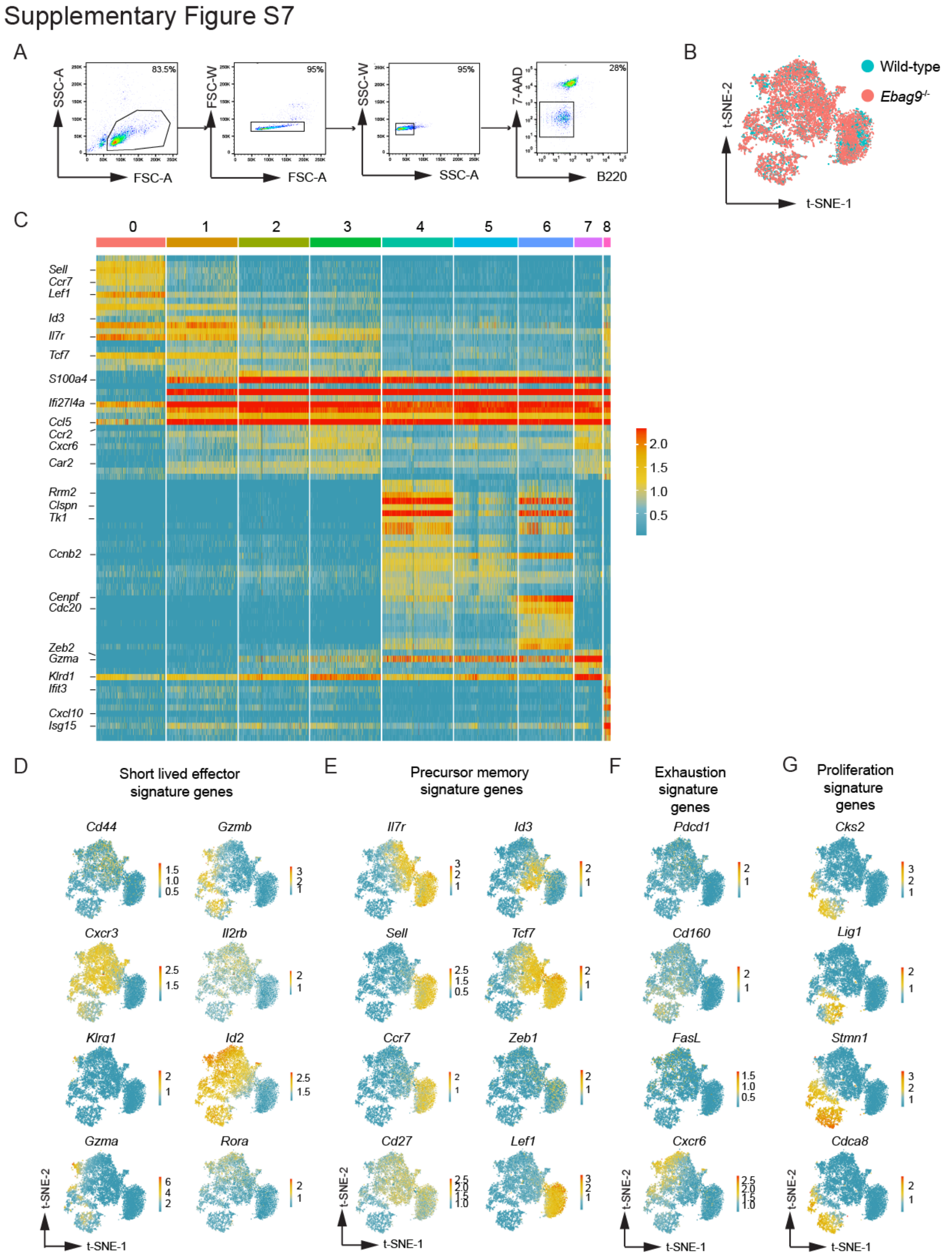

Figure S7: Single-cell transcriptomics of CD8$^+$ T cells responding to a non-inflammatory priming process distinguishes memory precursor cells from Wt and *Ebag9*$^{-/-}$ mice. (**A**) Gating strategy for FACS sorting of antigen-specific CD8$^+$ T

cells. This dot plot representation refers to Figure 6B. Frequencies are indicated as numbers on the gates. (**B**) t-SNE embeddings of merged Wt (n = 4301 cells) and *Ebag9*$^{-/-}$ (n = 6938 cells) antigen-specific CD8^{+} T cell profiles colored by Wt and *Ebag9*$^{-/-}$ status. **(C**) Heatmap displaying the top 10 genes expressed in each cluster as defined in Figure 6C. The columns correspond to the cells and the rows correspond to the genes. Cells are grouped by clusters. The color scale is based on a z-score distribution in the range indicated. **(D-G**) t-SNE projections displaying the color-coded expression of selected examples of genes associated with an effector (**D**), memory (**E**), exhausted (**F**), or proliferative (**G**) phenotype.

Table S1. Exhaustion marker status in $CD3^+CD8^+$ $CD44^{high}$ T cells derived from leukemia bearing mice. Data are given for LAG3 as geometric mean fluorescence intensity (gMFI).

	Naïve	*Eμ-Tcl1*x*Ebag9*$^{+/+}$	*Eμ-Tcl1*x*Ebag9*$^{-/-}$	Activated $CD8^+$ T cells[A]
	48,6	52	42,8	3495
		52	53,8	
	61,1	161	130	
		47,9	87,8	
	88,8	86,3	78,9	
		81,3	117	
	18	31,9	45,7	
		85,1	51,9	
	36,8	34,8	22,4	
		36,5	6,3	
Mean	50,7	66,9	63,7	

[A] splenocytes from naive control C57.BL/6 mouse; *in vitro* stimulated for 2 days by anti-CD3 and anti-CD28 plate-bound antibodies.

Table S2. Exhaustion marker status in $CD3^+CD8^+$ $CD44^{high}$ T cells derived from leukemia bearing mice. Data are given for PD-1 as geometric mean fluorescence intensity (gMFI).

	Naïve	*Eµ-Tcl1*x*Ebag9*$^{+/+}$	*Eµ-Tcl1*x*Ebag9*$^{-/-}$	Activated $CD8^+$ T cells[A]
	72,1	70,4	61,9	731
		78,3	62,5	
	58,5	116	206	
		66,4	125	
	132	172	111	
		169	94	
	47,5	55,8	78,5	
		128	111	
		21	30,1	
			78,4	
Mean	77,5	97,4	95,8	

[A] splenocytes from naive control C57.BL/6 mouse; *in vitro* stimulated for 2 days by anti-CD3 and anti-CD28 plate-bound antibodies.

Supplementary Material and Methods

Material

Table S3. Reagents.

Reagent or Resource	Source	Identifier
**Antibodies**		
Ultra-LEAF Purified anti-mouse CD3 (clone: OKT3)	Biolegend	Cat # 317326<br>RRID:AB_11150592
Ultra-LEAF Purified anti-mouse CD28 (clone: CD28.2)	Biolegend	Cat # 102116<br>RRID:AB_11147170
Purified anti-mouse CD16/32 (clone: 93)	Biolegend	Cat# 101302<br>RRID: AB_312801
PB Rat anti-mouse CD8 (clone: KT15)	ProImmune	Cat# A502-3A
PB Rat anti-mouse CD8 (clone: KT15)	Bio Rad	Cat# MCA609PBT<br>RRID: AB_2260086
PE Pro 5 MHC Pentamer H2Db	ProImmune	F327-2A-E
PE MHC Dextramer H2Db	Immudex	JA2189-PE
PE Pro 5 MHC Pentamer H2Kb	ProImmune	F562-2A-E
PE MHC Dextramer H2Kb	Immudex	JD4163-APC
PE Rat anti-mouse CD8 (clone: 53-6.7)	Biolegend	Cat# 100707<br>RRID: AB_312746
APC Rat anti-mouse CD8 (clone: 53-6.7)	Biolegend	Cat# 100712<br>RRID: AB_312751
PerCP Rat anti-mouse/ human CD44 (clone: IM7)	Biolegend	Cat# 103036<br>RRID: AB_10645506
APC Rat anti-mouse/ human CD44 (clone: IM7)	Biolegend	Cat# 103012<br>RRID: AB_312963
FITC Rat anti-mouse CD19 (clone: 6D5)	Biolegend	Cat# 115506<br>RRID: AB_313641
PE Rat anti-mouse CD19 (clone: 6D5)	Biolegend	Cat# 115508<br>RRID: AB_313643
FITC Syrian hamster anti-mouse/ human KLRG1 (clone: 2F1/ KLRG1)	Biolegend	Cat# 138410<br>RRID: AB_10643582
FITC Rat anti-mouse CD127 (clone: A7R34)	Biolegend	Cat# 135007<br>RRID: AB_1937231
PE Rat anti-mouse CD127 (clone: A7R34)	Biolegend	Cat# 135009<br>RRID: AB_1937252
FITC Armenian hamster anti-mouse/ human/ rat CD27 (clone: LG.3A10)	Biolegend	Cat# 124207<br>RRID: AB_1236463
APC Mouse anti-mouse CD45.2 (clone: 104)	Biolegend	Cat# 109814<br>RRID: AB_389211
AF488 Mouse anti-mouse CD45.2 (clone: 104)	Biolegend	Cat# 109816<br>RRID: AB_492868
PerCP Mouse anti-mouse CD45.1 (clone: A20)	Biolegend	Cat# 110726<br>RRID: AB_893345
FITC Mouse anti-mouse CD45.1 (clone: A20)	Biolegend	Cat# 110705<br>RRID: AB_313494
FITC Mouse anti-mouse CD90/ CD90.1 (clone: OX7)	Biolegend	Cat# 202503<br>RRID: AB_314014

APC Mouse anti-mouse CD90/ CD90.1 (clone: OX7)	Biolegend	Cat# 202526<br>RRID: AB_1595470
APC Rat anti-mouse CD90.2 (clone: 30-H12)	Biolegend	Cat# 105311<br>RRID: AB_313182
PE Hamster anti-mouse Vβ8.3 (clone: 1B3.3)	BD	Cat# 553664
APC Rat anti-mouse CD4 (clone: GK1.5)	Biolegend	Cat# 100412<br>RRID: AB_312697
PB Rat anti-mouse CD4 (clone: GK1.5)	Biolegend	Cat# 100428<br>RRID: AB_493647
APC Rat anti-mouse CD4 (clone: RM4-5)	Biolegend	Cat# 100516<br>RRID: AB_312719
PerCP Rat anti-mouse CD25 (clone: PC61)	Biolegend	Cat# 102027<br>RRID: AB_893290
AF488 Rat anti-mouse CD25 (clone: PC61)	Biolegend	Cat# 102018<br>RRID: AB_493334
FITC Rat anti-mouse CD62L (clone: MEL14)	Biolegend	Cat# 10441206<br>RRID: AB_313093
APC Rat anti-mouse CD62L (clone: MEL14)	Biolegend	Cat# 104412<br>RRID: AB_313099
PB Rat anti-mouse/ human CD45R/ B220<br>(clone: RA3-6B2)	Biolegend	Cat# 103227<br>RRID: AB_492876
PE Rat anti-mouse FOXP3 (clone: FJK-16s)	ebioscience	Cat# 12-5773-82<br>RRID: AB_465936
Mouse Vβ TCR Screening Panel	BD	Cat# 557004
PE Rat anti-mouse LAG-3 (clone C9B7W)	Biolegend	Cat#125208
PE Rat anti-mouse PD-1 (clone RMP1-30)	Biolegend	Cat#109103
FITC Rat anti-mouse IFN-γ (clone XMG1.2)	BD	Cat #562019
APC Rat anti-mouse TNF-α (clone MP6-XT22)	BD	Cat #554420
**Other Reagents**		
FOXP3 Transcription Factor Staining Buffer Set	ebioscience	00-5523-00
CytoFast Fix/ Perm Set	Biolegend	426803
FIX&PERM Cell Permeabilization Kit	Molecular Probes	Cat #GAS003
RPMI 1640 Medium	Gibco	21875-034
2-Mercaptoethanol	Gibco	31350-010
Penicillin/ Streptomycin 100x	Gibco	15070063
Sodium Pyruvate 100mM	Gibco	11360070
L-Glutamine 200 mM	Gibco	25030081
MEM Non Essential Amino Acids 100x	Gibco	11140035
Fetal Bovine Serum	Gibco	26140079
Recombinant Murine Il-2	Peprotech	212-12
Recombinant Murine Il-7	Peprotech	217-17
CD8a+ T cell Isolation Kit, mouse	Miltenyi	130-104-075
LS columns	Miltenyi	130-042-401
Cytotox 96 Non Radioactive Cytotoxicity Assay	Promega	G1781
5,5`-Dithio-*bis*-(2-nitrobenzoic acid)	Sigma Aldrich	Cat #**D8130**
N^{α}-Benzyloxycarbonyl-L-lysine Thiobenzyl Ester	Merck Millipore	Cat #200274
LegendPlex Murine Inflammatory Panel, Cytokine Bead Array	Biolegend	Cat #740446

Methods

Secretion assay

This assay was done essentially as described (1).

Naive $CD8^+$ T cells were stimulated by culturing splenocytes for 3 days in complete RPMI1640 medium supplemented with 10% FCS, β-mercaptoethanol, penicillin-streptomycin, glutamine, Na-pyruvate, and non-essential amino acids with plate-bound anti CD3ε mAb (3 μg/ml) and anti-CD28 mAb (2 μg/ml) in the presence of 20 U/ml IL-2 (Peprotec). $CD8^+$ T cells were pooled from 2-3 animals per assay, and then purified by negative selection with MACS beads (Miltenyi Biotec). CTLs were then further cultured for another 2 days in the presence of IL-2 and IL-7 (Peprotec, 50U/ml). At day 5-6, CTLs ($2x10^5$ cells/well, in 100 μl) were transferred into flat-bottom 96-well plates coated with 3 μg/ml anti-CD3 and 2 μg/ml anti-CD28. As a control for basal secretion, plates were left uncoated. Cell supernatant was harvested after 4 hrs and enzymatic activity for granzyme A and β-hexosaminidase was determined, as described previously (1). Briefly, Granzyme A activity was determined by cleavage of the substrates N^{α}-Benzyloxycarbonyl-L-lysine Thiobenzyl Ester, Hydrochloride and 5,5´-Dithio-bis-2-nitrobenzoic acid (DTNB). Photometric detection at 405 nm was performed. For determination of β-hexosaminidase activity, the cleavage of the substrate 4-Methylumbelliferyl-2-acetoamido-2-desoxy-β-D-glucopyranosid was analyzed by spectral photometric measurement at 355 and 450 nm. Granzyme B activity was measured by a colorimetric assay where the substrate Ac-IEPD-pNA (N-Acetyl-ILE-GLU-PRO-ASP-p-Nitroanilide) is cleaved and can be detected using an ELISA reader at 405 nm, exactly as described by the manufacturer (Sigma A6470). Cell lysates were generated to determine total enzymatic activity and

the enzymatic activity in the supernatant (release %) was calculated as relative to the total.

In vivo killing assay

Female Wt and Ebag9$^{-/-}$ mice were immunized twice i.p. with 5x10^{6} male (HY^{+}) splenocytes (day 0 and 14). At the indicated times after the first immunization, the *in vivo* killing assay was performed. For that, donor splenocytes from naïve HY^{-} female and HY^{+} male mice were isolated and labeled with different amounts of eFluor-670 for 10 min at 37°C. HY^{+} male splenocytes were stained with 1 μM eFluor-670, while HY^{-} female splenocytes were stained with 0.1 μM eFluor-670. After washing, 2x10^{7} stained HY^{+} male and HY^{-} female splenocytes were injected i.v. into immunized mice at 1:1 ratio. After 22 h, mice were sacrificed; splenocytes were isolated and analyzed by flow cytometry for eFluor-670. As a naïve control, non-immunized mice were included in each assay. To calculate the specific lysis, the following formula was applied:

% *in vivo* killing = [1-(control ratio/experimental ratio)]x100

Ratio = % low eFluor670 peak/% high eFluor670 peak

(Low = HY^{-}, high = HY^{+}; control = naïve, experimental = immunized mice)

Proliferation assay

This assay was done essentially as described (1). Activated T cells against the HY-antigen were generated in a standard mixed lymphocyte reaction (MLR). Briefly, CTLs were stimulated by coculture of 4x10^{6}/ml female splenocytes from the effector strains (B6) Wt and *Ebag9*$^{-/-}$ with 4x10^{6}/ml (B6) irradiated (3,000 rads) male splenocytes as stimulators in complete RPMI1640 medium. After 5 days, CTLs were

purified by CD8 MACS bead separation. $CD8^+$ lymphocytes were then titrated and cultured for 4 hrs. A Cell Titer96 Aqeous non-radioactive cell proliferation assay (Promega) was employed, where the conversion of a tetrazolium salt into a formazan product in cell culture was recorded as the absorbance at 490 nm.

Single cell RNA sequencing (scRNA-seq)

FACS-sorted $CD8^+$ T cells were counted and quality checked before loading each sample on individual lane using a Chromium controller (10X Genomics). Processing was done following 10x Chromium Single Cell 3'–end Reagent Kit User Guide (v3.1 Chemistry, 10X Genomics). Libraries were sequenced using paired-end sequencing (28nt read 1 and 91nt read 2) with a single sample index (8nt) on the Illumina Novaseq 6000. We used Cell Ranger (version 3.1.0, 10X Genomics) with genome build mm10 (Ensembl, GRCm38.93) for alignment, filtering, UMI counting, barcode counting and gene-barcode matrix generation. To remove potential ambient RNAs, we used the remove-background function in CellBender (version 1) (https://www.biorxiv.org/content/10.1101/791699), which removes ambient RNA contamination and random barcode swapping from the raw UMI-based data. Data integration, cell clustering, and dimension reduction were performed using Seurat (version 3.1.5) with SCTransform (2). Filtering was conducted by retaining cells that had features detected greater than 300 and only those features were considered that were detected in at least 5 cells. Removing non-T cells from the data set was guided by gene signature scores from blacklisted gene sets (3). Percent mitochondrial content and cell-cycle scores were used to regress out unwanted sources of variation (4). For dimensionality reduction, 2000 highly variably expressed genes were summarized by principle component analysis (PCA), and the first 10 principle components were

further summarized using t-distributed stochastic neighbor embedding (tSNE) dimensionality reduction (5). Further visualizations were achieved using ggplot2 functions (https://ggplot2.tidyverse.org) mostly accessible in https://shiny.mdc-berlin.de/sivisca/. RNA velocity analysis was performed using mapped reads with velocyto (version 0.17.17) (6) and scVelo (version 0.2.2) (7). Single-cell data have been deposited in NCBI's Gene Expression Omnibus (8) and are accessible through GEO Series accession number GSE156611 (https://www.ncbi.nlm.nih.gov/geo/query/acc.cgi?acc= GSE156611).

References

1. Ruder, C., Hopken, U.E., Wolf, J., Mittrucker, H.W., Engels, B., Erdmann, B., Wollenzin, S., Uckert, W., Dorken, B., and Rehm, A. 2009. The tumor-associated antigen EBAG9 negatively regulates the cytolytic capacity of mouse CD8+ T cells. *J Clin Invest* 119:2184-2203.
2. Stuart, T., Butler, A., Hoffman, P., Hafemeister, C., Papalexi, E., Mauck, W.M., 3rd, Hao, Y., Stoeckius, M., Smibert, P., and Satija, R. 2019. Comprehensive Integration of Single-Cell Data. *Cell* 177:1888-1902 e1821.
3. Szabo, P.A., Levitin, H.M., Miron, M., Snyder, M.E., Senda, T., Yuan, J., Cheng, Y.L., Bush, E.C., Dogra, P., Thapa, P., et al. 2019. Single-cell transcriptomics of human T cells reveals tissue and activation signatures in health and disease. *Nat Commun* 10:4706.
4. Yim, A., Koti, P., Bonnard, A., Marchiano, F., Durrbaum, M., Garcia-Perez, C., Villaveces, J., Gamal, S., Cardone, G., Perocchi, F., et al. 2020. mitoXplorer, a visual data mining platform to systematically analyze and visualize mitochondrial expression dynamics and mutations. *Nucleic Acids Res* 48:605-632.
5. Kobak, D., and Berens, P. 2019. The art of using t-SNE for single-cell transcriptomics. *Nat Commun* 10:5416.
6. La Manno, G., Soldatov, R., Zeisel, A., Braun, E., Hochgerner, H., Petukhov, V., Lidschreiber, K., Kastriti, M.E., Lonnerberg, P., Furlan, A., et al. 2018. RNA velocity of single cells. *Nature* 560:494-498.
7. Bergen, V., Lange, M., Peidli, S., Wolf, F.A., and Theis, F.J. 2020. Generalizing RNA velocity to transient cell states through dynamical modeling. *Nat Biotechnol* 38:1408-1414.
8. Edgar, R., Domrachev, M., and Lash, A.E. 2002. Gene Expression Omnibus: NCBI gene expression and hybridization array data repository. *Nucleic Acids Res* 30:207-210.
